# Redox Potentials of Disulfide Bonds in LOXL2 Studied by Nonequilibrium Alchemical Simulation

**DOI:** 10.3389/fchem.2021.797036

**Published:** 2021-12-14

**Authors:** Lirui Lin, Haiying Zou, Wenjin Li, Li-Yan Xu, En-Min Li, Geng Dong

**Affiliations:** ^1^ Department of Biochemistry and Molecular Biology, Shantou University Medical College, Shantou, China; ^2^ Medical Informatics Research Center, Shantou University Medical College, Shantou, China; ^3^ Key Laboratory of Molecular Biology in High Cancer Incidence Coastal Area of Guangdong Higher Education Institutes, Shantou University Medical College, Shantou, China; ^4^ Institute for Advanced Study, Shenzhen University, Shenzhen, China; ^5^ Cancer Research Center, Shantou University Medical College, Shantou, China

**Keywords:** LOXL2, disulfide bond, redox potential, non-equilibration simulation, alchemical method

## Abstract

Lysyl oxidase-like 2 (LOXL2) is a metalloenzyme that catalyzes the oxidative deamination ε-amino group of lysine. It is found that LOXL2 is a promotor for the metastasis and invasion of cancer cells. Disulfide bonds are important components in LOXL2, and they play a stabilizing role for protein structure or a functional role for regulating protein bioactivity. The redox potential of disulfide bond is one important property to determine the functional role of disulfide bond. In this study, we have calculated the reduction potential of all the disulfide bonds in LOXL2 by non-equilibrium alchemical simulations. Our results show that seven of seventeen disulfide bonds have high redox potentials between −182 and −298 mV and could have a functional role, viz., Cys573–Cys625, Cys579–Cys695, Cys657–Cys673, and Cys663–Cys685 in the catalytic domain, Cys351–Cys414, Cys464–Cys530, and Cys477–Cys543 in the scavenger receptor cysteine-rich (SRCR) domains. The disulfide bond of Cys351–Cys414 is predicted to play an allosteric function role, which could affect the metastasis and invasion of cancer cells. Other functional bonds have a catalytic role related to enzyme activity. The rest of disulfide bonds are predicted to play a structural role. Our study provides an important insight for the classification of disulfide bonds in LOXL2 and can be utilized for the drug design that targets the cysteine residues in LOXL2.

## Introduction

Lysyl oxidase like 2 (LOXL2) is a copper-dependent amine oxidase enzyme, which belongs to the lysyl oxidase (LOX) family (Smith-Mungo and Kagan, 1998; Jourdan-Le Saux et al., 1999; Csiszar, 2001). It catalyzes the oxidation of collagen and elastin to promote cross-linking, leading to the stiffening of the extracellular matrix (ECM) (Smith-Mungo and Kagan, 1998). Aside from its basic enzyme function, LOXs are found to be significant to human diseases, e.g., several types of cancer (Kagan, 2000; Barker, Cox, and Erler, 2012; Johnston and Lopez, 2018). The members of the LOX family have complex and paradoxical roles of both tumor suppressor and metastasis promoter (Barker, Cox, and Erler, 2012; Johnston and Lopez, 2018). For LOXL2, it is highly expressed in tumors (Barry-Hamilton et al., 2010) and proposed to act as a metastasis promoter (Kirschmann et al., 2002; Peinado et al., 2008; Barry-Hamilton et al., 2010). Thus, many studies are targeting LOXL2 to inhibit the metastasis/invasion of cancer (Hutchinson et al., 2017; Chopra et al., 2020; Klepfish et al., 2020).

**FIGURE 1:**
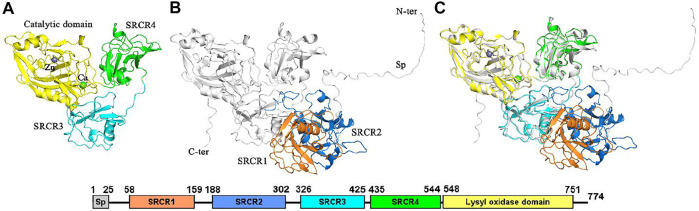
The structure of LOXL2. **(A)** The x-ray structure (PDB ID: 5ZE3). **(B)** Structure from Alphafold2. **(C)** Alignment of the two structures (RMSD = 0.62 Å). Sp: signal peptide. Bound zinc and calcium are displayed as spheres. All structure figures are prepared using PyMOL (Schrödinger, 2010).

LOXL2 is synthesized as an 87-kDa proenzyme of 774 amino acids. In 1997, the LOXL2 gene was recognized as a reduced transcript in kinds of non-adherent tumor cell lines compared to adherent tumor cell lines (Saito et al., 1997). LOXL2 has a conserved catalytic region that contains a copper-binding domain as well as a quinone cofactor, which is formed by highly conserved lysine and tyrosine residues. There are four scavenger receptor cysteine-rich (SRCR) domains linking to the catalytic domain. The crystal structure of human LOXL2 in a precursor state was first determined in 2018 (Zhang et al., 2018), but SRCR1 and SRCR2 domains are missing (Figure 1A). In addition, the catalytic center is occupied by a zinc ion instead of a copper ion. Recently, the complete structure of LOXL2 is published in the AlphaFold Protein Structure Database (AlphaFold) (Figure 1B), which is predicted by AlphaFold2 (Jumper et al., 2021; Bershtein et al., 2021). It can be seen from the alignment of the X-ray structure and AlphaFold structure that the predicted structure has a good overlap with the experimental structure with RMSD of 0.62 Å (Figure 1C). Notably, the SRCR3 and SRCR4 domains belong to the group A of the SRCR family that is characterized by a conserved pattern by three disulfide bonds and a single α-helix surrounded by β-strands (Hohenester et al., 1999). In addition, there are 5 disulfide bonds existing in the catalytic domain. As a whole, the crystal structure of LOXL2 presents a triangular shape, i.e., SRCR3, SRCR4, and the catalytic domains are located at three vertexes of the triangle, respectively (Zhang et al., 2018). The function of SRCR domains is deduced to mediate homotopical or heterotypical protein–protein interaction in the extracellular matrix. Heretofore, we know that most proteins with the SRCR domain always act as extracellular pattern recognition receptor (Martinez et al., 2011). However, for LOXL2, the biological functions of SRCR domains are still not fully understood.

**FIGURE 2:**
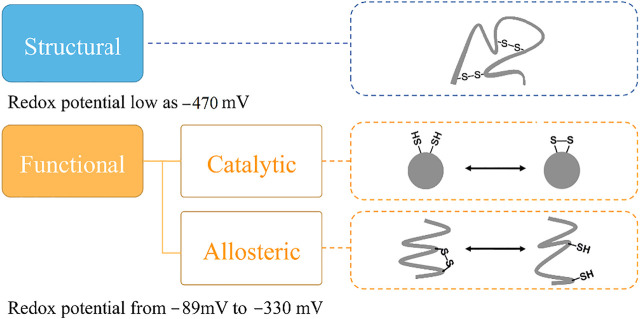
The classification of disulfide bonds. Reduction potential of the disulfide bond is one key indicator to classify its biological role in proteins. The disulfide bond with a structural role simply maintains the structure of protein. For disulfide bond with an allosteric role, it can alter the substrate protein structure to regulate protein function or activity by being cleaved, and the disulfide bond with a catalytic role performs the functional role without change in the substrate protein structure.

It is interesting that all the cysteine residues are cross-linked to form disulfide bonds in LOXL2. Three disulfide bonds are formed in each SRCR domain and five in the catalytic domain, which gives seventeen disulfide bonds totally. It has been validated that protein disulfide bonds have two roles for proteins: one is to maintain the structure of the protein, and the other one is to modulate the function of protein (Wouters et al., 2010) (Figure 2). Furthermore, there are two types of functional bonds, i.e., catalytic and allosteric function (Cook and Hogg, 2013; Cook et al., 2013; Butera et al., 2014). The catalytic bonds are found at the active sites of enzymes that mediate thiol/disulfide exchange in other proteins, i.e., oxidoreductases (Berndt et al., 2008). Allosteric disulfide bonds are defined to regulate the manner in which proteins act its function by breaking or forming in a precise way (Hogg, 2009) and by mediating a change when they are reduced or oxidized (Hogg, 2003; Schmidt et al., 2006). The redox state of allosteric disulfide bonds is controlled by oxidoreductase (Wong and Hogg, 2011). In addition, oxidoreductases will only cleave a disulfide bond with a relatively low reduction potential, meaning that oxidoreductases do not cleave structural bonds (Hogg, 2009). Thus, the formation of functional disulfide bond is an important factor in regulating the molecular mechanism of the protein (Zhou et al., 2010; Butera et al., 2018).

Targeting the functional disulfide bond is one of the methods to develop new drugs for treating diseases (Barry-Hamilton et al., 2010; Rodriguez et al., 2010; Benson et al., 2017; Hecht et al., 2017; Harrison et al., 2018; Muir et al., 2019). For this purpose, it is important to distinguish the functional bond from the structural one. One important method to determine the role of the disulfide bond is based on redox potential. Previous studies have shown that structural protein disulfide bonds normally have low reduction potentials, whereas high reduction potentials are usually measured in functional bonds (Gilbert, 1990; Wunderlich and Glockshuber, 1993; Schmidt et al., 2006; Cook, 2019). Reduction potentials for functional disulfide bonds (including oxidoreductases) range from −89 to −330 mV (Gilbert, 1990; Wunderlich and Glockshuber, 1993; Chen and Hogg, 2006; Wouters, Fan, and Haworth, 2010). Structural disulfide bonds typically have reduction potentials < −470 mV (Gilbert, 1990), shown in Figure 2.

To determine the redox potential of the disulfide bond in protein, maleimide-biotin (MPB) labeling of free cysteine thiols and Western blot densitometry are suitable experimental ways (Liang et al., 2011; Cook et al., 2013; Chiu et al., 2014), and differential cysteine labeling, tandem mass spectrometry, and the structural character of the disulfide bond are also used as references in determining the function role of the disulfide bond (Bekendam et al., 2016; Read et al., 2017; Butera et al., 2018). Because of the fact that redox potential is very sensitive to the structure of protein, the pKa value of sulfide, the protein’s electrostatic environment, etc., a stable measurement method for redox potential is demanding (Quan et al., 2007; Jensen and Li, 2009; Li et al., 2015). Redox potentials can also be calculated by theoretical methods, e.g., molecular dynamics (MD) simulations (Pohorille et al., 2010; Dellago and Hummer, 2013; Li et al., 2015) and quantum mechanics and molecular mechanics (QM/MM) approaches (Blumberger, 2008; Kamerlin et al., 2009; Zeng et al., 2009; Baldus and Gräter, 2012). A limitation of the QM/MM approaches is that the only small part of the MM region is allowed to be optimized, so QM/MM calculation might not be an optimal way for flexible proteins. For LOXL2, the disulfide bond formed by cystine residues might affect the structure and function by allosteric effect, which means that the conformation of the protein would change much due to the formation/cleavage of the disulfide bond. For the MD simulation approach, it is found that the predicted redox potentials after scaling have a small deviation of 8 kJ/mol (43 mV) in our previous study (Li et al., 2015), and this method is an efficient way to study the reduction potential of the disulfide bond.

In this paper, we set up the entire structure of LOXL2 based on the crystal structure and AlphaFold-predicted structure. Then, MD simulations were carried out to equilibrate the system. Finally, non-equilibrium free energy calculations were performed to calculate the reduction potential of the seventeen disulfide bonds in LOXL2, i.e., mimicking the transformation from oxidized to reduced state reversibly. Five hundred replicas of transformation are performed to get the reduction potential. Our results show that seven disulfide bonds are predicted to play a functional role, i.e., one allosteric role of the disulfide bond in SRCR3, and two and four disulfide bonds with a catalytic role in the SRCR4 and catalytic domains, respectively. The other disulfide bonds are predicted to have a structural role.

## Methods

### System Setup

**FIGURE 3:**
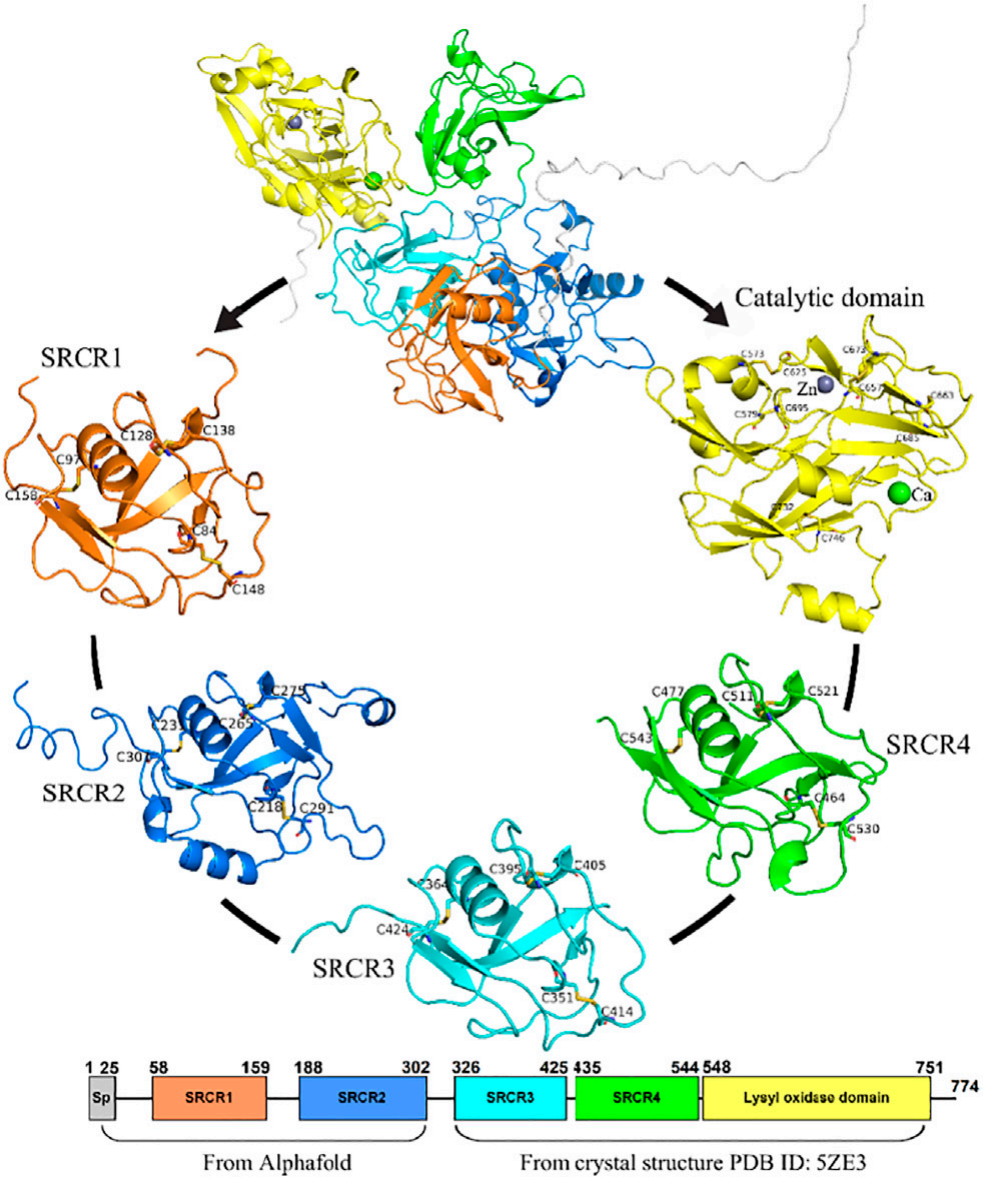
The five systems of LOXL2 in MD simulations for the SRCR 1-4 and catalytic domains. Disulfide bonds are shown by sticks. Bound zinc and calcium are displayed as spheres.

In this study, the structure of LOXL2 was divided into 5 systems (In Figure 3): SRCR1−4 and catalytic domains. The structures of SRCR1 and 2 domains were predicted by AlphaFold2 in the AlphaFold Protein Structure Database (Bershtein et al., 2021; Jumper et al., 2021), and the remaining parts were taken from the 2.60-Å resolution of X-ray crystal structure (PDB ID: 5ZE3) (Huang et al., 2018). Systems 1–5 include residues 50–165, 166–321, 322–431 and 432–546, and 547–762, respectively.

The protonation states of all the residues are determined by using the pKa value from PROPKA (Olsson et al., 2011), a comprehensive evaluation for solvent accessibility, hydrogen-bond pattern around His residues, and possible formation of ionic pairs. All Arg, Lys, Asp, and Glu residues were assumed to be charged. Among the His residues, His 67, 91, 94, 221, 271, 623, and 626 were protonated on the NE2 atom, and His 604, 628, 630, 652, and 739 were assumed be protonated on ND1, whereas the remaining His residues were modeled as doubly protonated.

All systems were solvated in a cubic box of TIP3P water with a minimum distance of 12 Å from protein structures to the box boundary by using the *genbox* module in GROMACS-4.5.5 (Jorgensen et al., 1983; Hess et al., 2008; Gillan et al., 2016). A salt concentration of 0.1 mol/l was used to mimic physiological conditions and to neutralize the systems.

### MD Simulation

All MD simulations were carried out using the GROMACS-4.5.5 package (Hess et al., 2008). Temperature was kept at 300 K by using velocity rescaling (Grest and Kremer, 1986; Bussi et al., 2007); Pressure was kept at 1 bar by using isotropic pressure coupling with Parrinello–Rahman barostat and a coupling constant of 1.0 ps (Parrinello and Rahman, 1981). For all systems, energy minimization was first performed by using the steepest descent algorithm. Next, a 100-ps NVT simulation with restraints is performed for heavy atoms of protein using a spring constant of 1,000 kJ/(mol·nm^2^) (Hess, 2008). Finally, a 50-ns production simulation in the NPT ensemble was performed. Sampling structures were saved every 25 ps, which gave 2,000 structures for each system. Non-bonded interactions were calculated within a cutoff of 1.0 nm. Electrostatic interactions beyond 1.0 nm were treated with particle-mesh Ewald (PME) with a grid spacing of 0.12 nm (Essmann et al., 1995). The periodic boundary condition was used for all simulations. In addition, for the catalytic system, two metal ions CA^2+^ and Zn^2+^ and their coordinated residues were restrained at crystal structure positions. All amino acids were described by the CHARMM27 force field (Bjelkmar et al., 2010).

### Non-Equilibrium Free Energy Calculation

**FIGURE 4:**
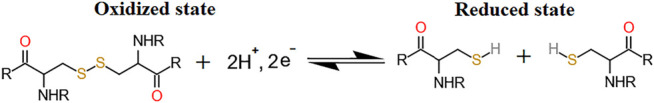
Redox reaction of disulfide bond formed by two cysteines.

To estimate the free energy differences between reduced and oxidized states in the redox reaction, non-equilibrium transition was constructed based on the previous study (Pohorille et al., 2010; Li et al., 2015; Gräter and Li, 2019). As is shown in Figure 4, the disulfide bond is formed in the oxidized state, and two normal Cys residues present the reduced state. For the two end states, 0.5-ns NVT equilibrium simulations are carried out, followed by 50-ns NPT production simulations. Subsequently, 500 snapshots were extracted every 55 ps from each production trajectory (the first 20 ns from the trajectories were discarded). Finally, 2 ns × 500 replicas of non-equilibrium simulations (backward and forward directions, Δλ = 0.00002 fs) were carried out to alchemically morph between the oxidized and reduced states. Free energy differences were calculated by the *pmx* program (Gapsys et al., 2015), in which the work values were used to estimate the free energy differences basing on the Crooks fluctuation theorem (Crooks, 1999) and utilizing the Bennett acceptance ratio as a maximum likelihood estimator (Shirts et al., 2003).

**FIGURE 5:**
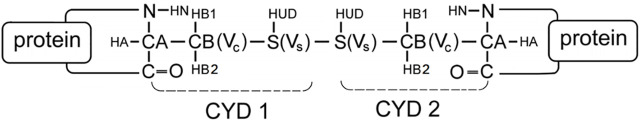
The scheme of CYD topology. CYD represents a disulfide-bonded cysteine residue that is expanded by one atom (HUD), which will be changed into a hydrogen; two virtual sites (V_c_ and V_s_, shown in the figure) and those are constrained to the same positions as CB and S, respectively. A disulfide bond shaped by two CYD residues in a protein according to the scheme.

For the transformation between cystine and two free cysteines, hybrid cysteine/cystine topologies were constructed based on our previous protocol (Gräter and Li, 2019). As is shown in Figure 5, a new cysteine residue type is added for the disulfide-bonded cysteine (CYS2), which is named residue CYD, and it includes one dummy atom HUD and two virtual sites (V_c_ and V_s_, respectively). Notably, the dummy atom and virtual sites do not have bonded and Lennard–Jones interactions, and its description is based on the CHARMM27 force field. A more detailed description for the parameters can be found in the previous studies (Gräter and Li, 2019).

**FIGURE 6:**
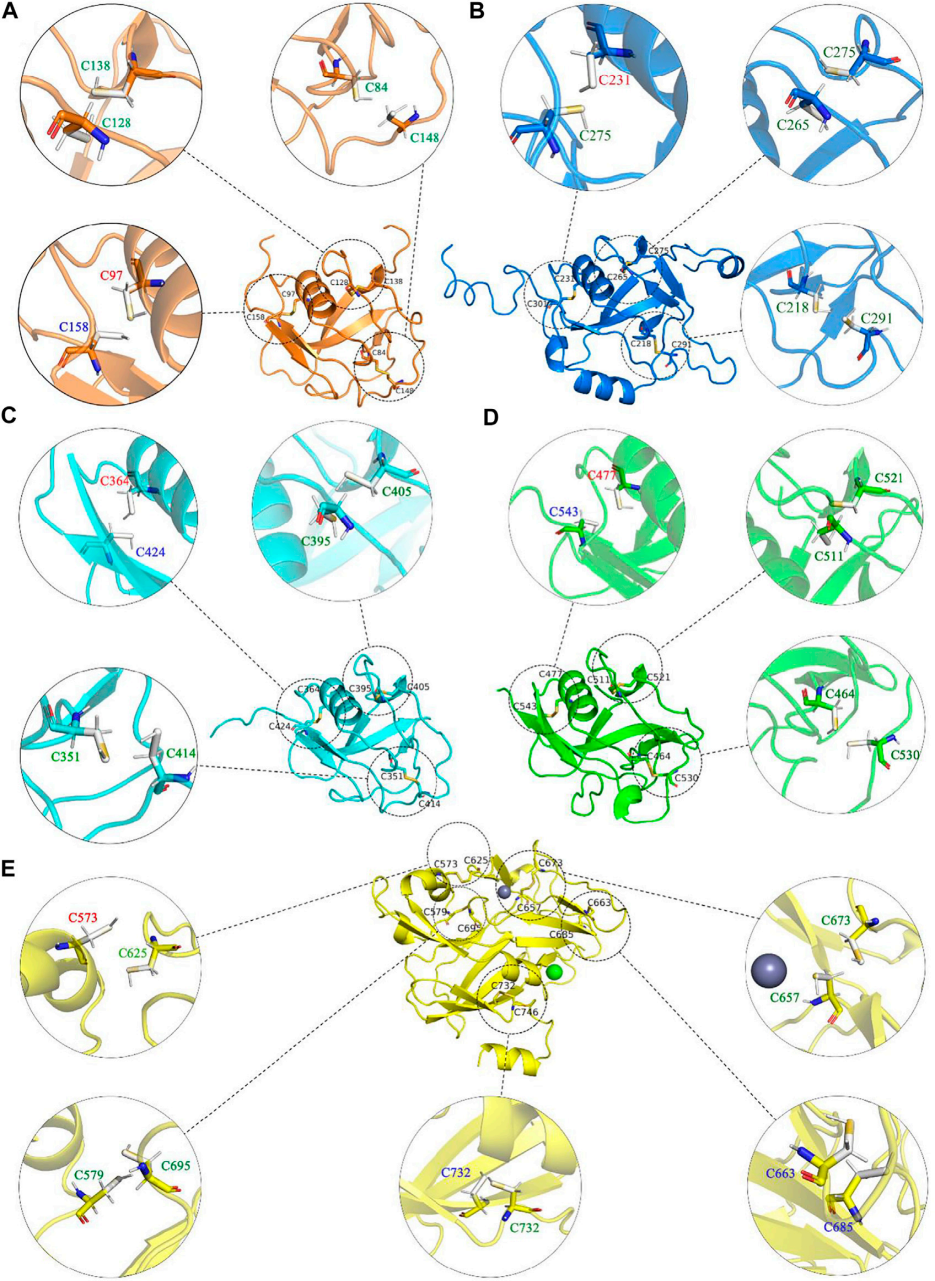
The oxidized and reduced states of each disulfide bond in LOXL2. **(A)**-E) show each domain in LOXL2, respectively. The dotted circles present oxidized state, solid circles present reduced state, and disulfide bond and Cys are shown as stick. The font color of residues stands for the secondary structure type of each disulfide bond connected: red: helix, blue: sheet, and greet: loop.

For alchemical MD simulations, a single topology approach was used (Gao et al., 1989; Axelsen and Li, 1998). When λ = 0 (oxidized state), there is no charge on the dummy atom of HUD and two virtual sites (V_c_ and V_s_), and no interaction between the dummy HUD atom and the two virtual sites. Thus, CYD is the same as CYS2 in the CHARMM27 force field. HUD is switched to a hydrogen atom and presents entire bonded interactions when λ = 1. Thus, CYD is a standard free cysteine residue. In addition, more bond interactions present in the oxidized state, i.e., the bond S1−S2, angles CB1−S1−S2, and related dihedral angles (subscripts 1 and 2 refer to the atoms in CYD1 and CYD2 in Figure 5). These bonded interactions are absent in two independent cysteine residues at λ =1. As is shown in Figure 6, we performed non-equilibrium transition for the seventeen disulfide bonds in LOXL2, and the oxidized and reduced states are presented. The disulfide bonds link three types of secondary structure of LOXL2, i.e., α helix, β sheet, and loop. The α helix and β sheet usually form a related conserved structure for specific function, while the loop is usually kept at a flexible state, which provides more space for interaction with adjacent areas (Pijning and Hogg, 2019).

### Redox Potential Calculation

Finally, the reduction potential (*E* ^*0*^) was calculated based on Nernst’s equation,

$$E^{0}=-\Delta G/nF$$

where ΔG is the free energy difference between the reduced and oxidized states in a redox reaction, *n* is the number of electrons transferred, and F is the Faraday constant. We use the Crooks Gaussian Intersection (CGI) method to calculate free energy differences between reduced and oxidized states in redox reactions. The method has been described in detail in the literature (Goette and Grubmuller, 2009).

## Results and Discussion

### The SRCR1 and SRCR2 Domains of LOXL2

For the LOX family, the crystal structure is only available for LOXL2, but without the SRCR1 and SRCR2 domains (Zhang et al., 2018). The structures of missing domains were predicted by AlphaFold2 (Bershtein et al., 2021; Jumper et al., 2021) and I-TASSA (Roy et al., 2010) servers. The structures from the three methods are compared with the crystal structure, and the AlphaFold2 structure was used in this study due to its smallest RMSD value of 0.62 Å (Figure 1; Supplementary Figure S1). Then, we constructed an entire system of LOXL2 by combining the crystal structure (PDB ID: 5ZE3) with the AlphaFold2 structure.

**FIGURE 7:**
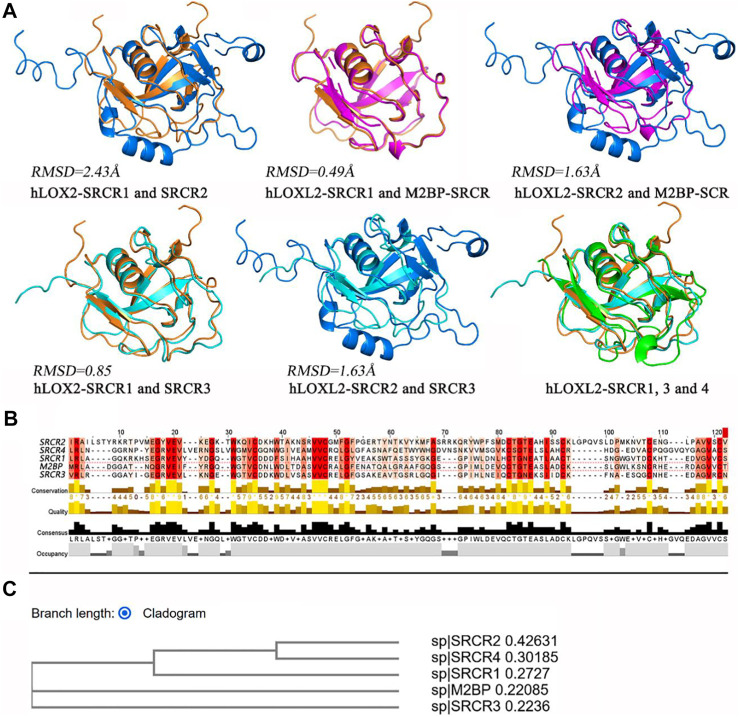
**(A)** Structure alignment for SRCR 1-4 of LOXL2 by using PyMOL (Schrödinger, 2010). **(B)** Multiple-sequence alignment by MUSCLE (Edgar, 2004). The depth of color indicates the conservation degree of residue, and the deeper color indicates more conservative. The histograms indicate the level in each character, score from 0 to 8. **(C)** Phylogenetic tree *via* cladogram rectangle by MUSCLE.

For the structures of SRCR domains in LOXL2, we make a multiple-sequence alignment by MUSCLE (Edgar, 2004) and a structure alignment by PyMOL (Schrödinger, 2010), visualized in Figure 7. In Figure 7A, the alignment of SRCR1 and SRCR2 presents an RMSD of 2.43 Å, and the RMSDs are respectively 0.49 and 1.63 Å when the SRCR1 and SRCR2 domains are aligned with M2BP, which is a standard SRCR of group A in the SRCR family (Hohenester et al., 1999). SRCR1 has almost the same structure with SRCR3 (RMSD = 0.85 Å). Overall, SRCR domains are highly conserved.

The sequence alignment results (Figures 5C, 7B) show the residue characters of SRCR 1-4, including conservation, quality, consensus, and occupancy evaluations. On account of the deep degree of cysteine, it can be seen that disulfide bonds have a high conservation degree. The phylogenetic tree *via* the cladogram rectangle shows the correlations between SRCR 1-4 and M2BP, in which SRCR domains 1 and 3 are closer to M2BP in the branch, whereas SRCR2 and 4 are at the third level. This might be one reason why RMSD values are large when the SRCR2 and SRCR4 domains aligned to M2BP. In this view, SRCR2 might inherit the function of SRCR1 in LOXL2, while evolving to get some new role compared to SRCR1. Accordingly, SRCR4 is evolved to be more versatile than SRCR3.

### Reduction Potentials for the Disulfide bonds in LOXL2

Disulfide bonds are formed by cysteines and have two roles in protein, i.e., one role is structural and the other one is functional (Cook, 2019). Previous studies demonstrated that the disulfide bonds with a structural role normally have a low reduction potential (<−470 mV), indicating that the bond is very stable and hard to be broken down. On the contrary, high reduction potentials (−89∼−330 mV) of disulfide bonds often present a functional role, and the bond distance is slightly longer than that of structural bonds (Cook, 2019; Pijning and Hogg, 2019). To distinguish the role of disulfide bonds in LOXL2, we calculated the reduction potential for all the disulfide bonds through nonequilibrium free energy simulations. In order to find a functional role of disulfide bonds, disulfide bonds with redox potentials in the range of −89∼−330 mV are deemed to have a functional role, whereas others are excluded to have a structural role.

According to our previous study, the reduction potentials have a correlation with experimental data, *E*_exp_ = 1.5 × *E*_cal_ −43 mV (Li, Baldus, and Gräter, 2015). We then added this correction to our results. All computational data are collected in Table 1. For the disulfide bonds of Cys351–Cys414, Cys464–Cys530, Cys477–Cys543, Cys573–Cys625, Cys579–Cys695, Cys657–Cys673, and Cys663–Cys685, they are predicted to be functional bonds. Furthermore, the remaining bonds are classified as structural bonds. It should be noted that two functions were found for LOXL2; i.e., one is the enzyme function related to amine oxidase activity, and the other one is the promoting effect of migration and invasion to cancer cells (Zou et al., 2020). The disulfide bond with a catalytic role is related to the enzymatic function of LOXL2, while the disulfide bond with an allosteric role is related to the function of migration and invasion in LOXL2 (Hogg, 2009; Friedl and Alexander, 2011).

**TABLE 1:** Data collection for the disulfide bonds in LOXL2.

Domain	Disulfide bond	Work	Reduction potential	Corrected reduction potential^a^
SRCR 1	Cys84–Cys148	37.20	−201.11	−348.67
SRCR 1	Cys97–Cys158	46.58	−249.13	−420.70
SRCR 1	Cys128–Cys138	36.22	−191.67	−334.51
SRCR 2	Cys218–Cys291	36.68	−192.07	−335.11
SRCR 2	Cys231–Cys301	49.78	−260.67	−438.00
SRCR 2	Cys265–Cys275	39.19	−205.22	−354.82
SRCR 3	Cys351–Cys414	28.59	−148.16	−269.24
SRCR 3	Cys364–Cys424	47.06	−243.87	−412.81
SRCR 3	Cys395–Cys405	37.63	−195.00	−339.51
SRCR 4	Cys464−Cys530	21.63	−112.09	−215.13
SRCR 4	Cys477–Cys543	28.66	−148.52	−269.78
SRCR 4	Cys511–Cys521	48.71	−252.84	−425.63
Catalytic	Cys573–Cys625	18.08	−93.69	−187.54
Catalytic	Cys579–Cys695	28.87	−149.61	−271.41
Catalytic	Cys657–Cys673	17.36	−89.96	−181.94
Catalytic	Cys663–Cys685	32.31	−167.44	−298.15
Catalytic	Cys732–Cys746	43.58	−225.84	−385.76

a Corrected reduction potential is *E*_corr_ = 1.5 × *E*_cal_−43 mV.

### Discussion of the Function Role of Disulfide Bonds in LOXL2

**FIGURE 8:**
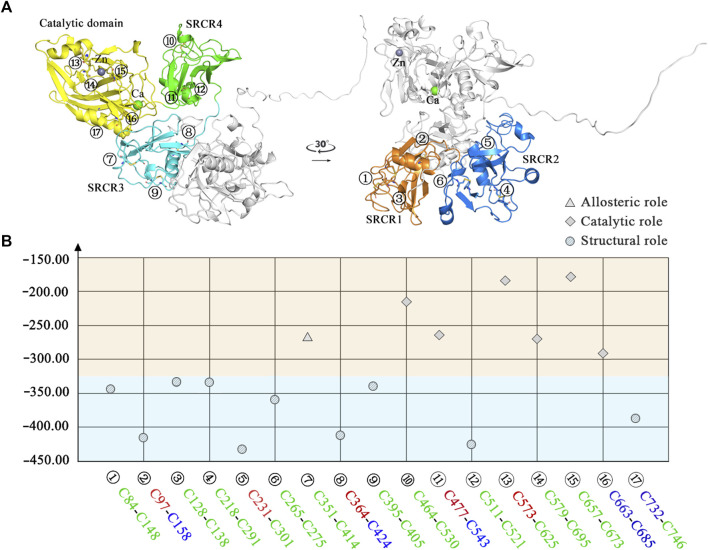
Classification of disulfide bonds in LOXL2. **(A)** The structure of LOXL2. **(B)** The reduction potential distribution of disulfide bonds. Blue area: structural function; orange area: allosteric or catalytic function. Icon for the role of disulfide bond: circle for structural role, triangle for allosteric role, and rhomb for catalytic role. The front color of the residue stands for the secondary structure type of each disulfide bond connected: red: helix, blue: sheet, and green: loop.

For the disulfide bonds in the SRCR 1 domains (①, ②, and ③ in Figure 8), all these three bonds have a reduction potential in the range of −335 to −420 mV, indicating that they have a structural role for LOXL2. Based on our previous study, LOXL2 without the SRCR1 domain (LOXL2 ΔSRCR1) has an approximate catalytic activity compared with wild-type LOXL2 (Zou et al., 2020). It is shown in Figure 8A that the SRCR1 domain is located far from the catalytic domain; it would be easy to understand that it owns less correlation to the catalytic reaction. On the other hand, LOXL2 ΔSRCR1 had little promotion effects on esophageal squamous cell carcinoma (ESCC) cellular mobility than wild-type LOXL2 in bioactive assay (Zou et al., 2020). Thus, it can be concluded that disulfide bonds of ①, ②, and ③ do not show an apparent effect to enzyme activity as well as the migration and invasion function, and they are structural disulfide bonds.

For the disulfide bonds in SRCR 2 domains (④, ⑤, and ⑥ as shown in Figure 8, the reduction potentials are lower than the threshold value −330 mV, and they are predicted to have a structural role. Similarly to LOXL2 ΔSRCR1, our previous bioactive assay showed that LOXL2 ΔSRCR2 does not change the oxidative activity and has no effects on ESCC cellular mobility (Zou et al., 2020). In addition, LOXL2 without SRCR1 and 2 shows indistinctive amine oxidase activity after proteolytic processing (Lopez-Jimenez et al., 2017), meaning that there might not be a functional role of disulfide bond in the SRCR1 and 2 domains of LOXL2. Thus, ④, ⑤, and ⑥ are evaluated to be structural disulfide bonds.

**FIGURE 9:**
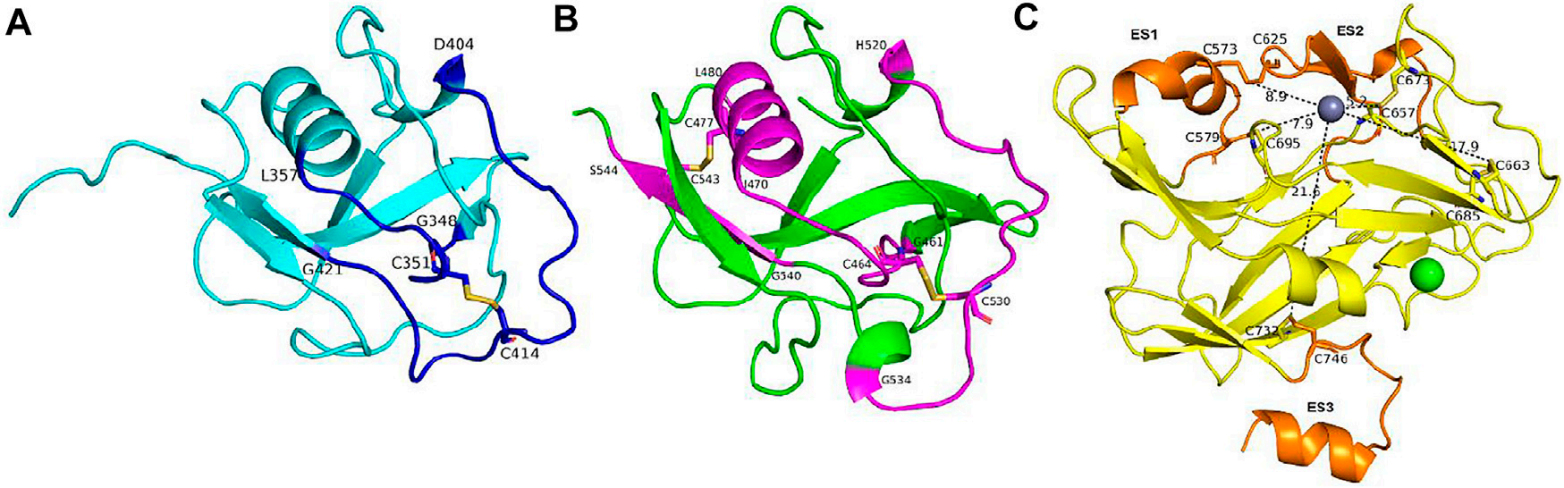
The structural character of disulfide bonds in the SRCR3-4 and catalytic domains. **(A)** and **(B)** The secondary structure of functional disulfide bonds linked in SRCR3 and SRCR4, colored by blue and purple, respectively. **(C)** The distances between catalytic center and the five disulfide bonds in the catalytic domain. Extra segments are colored orange; the rest of the conformations are colored yellow.

**FIGURE 10:**
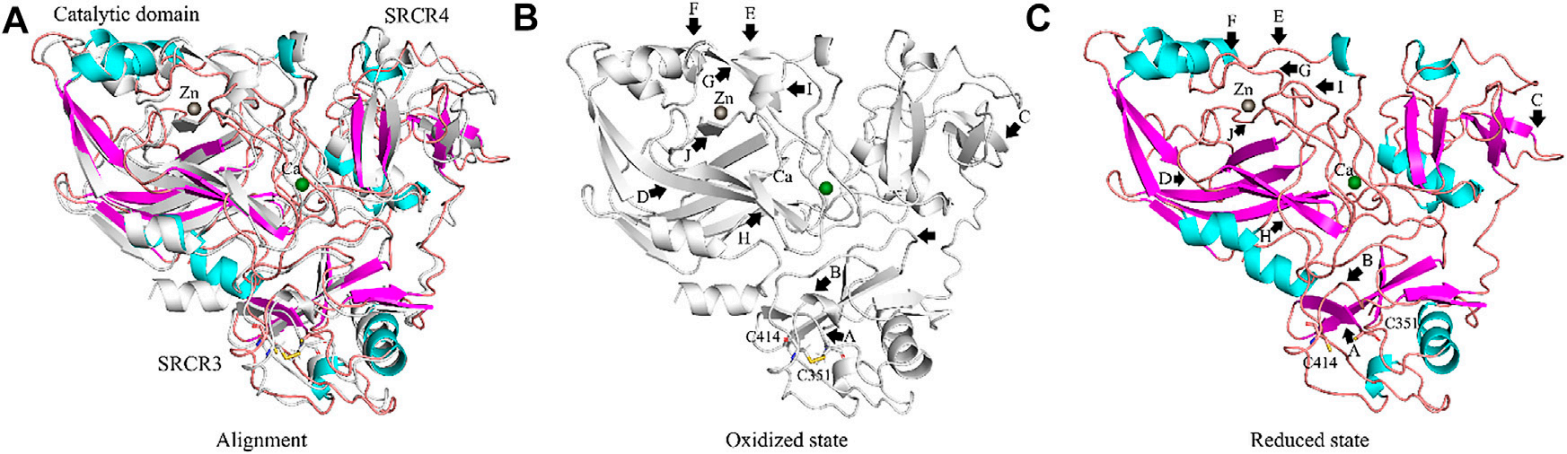
Conformation changes of LOXL2 from oxidized to reduced state of disulfide bonds Cys351–Cys414. **(A)** Alignment of LOXL2 at oxidized state (crystal structure of LOXL2, PDB ID: 5ZE3; Colored gray) and reduced state (after MD simulation; colored purple for α-helix, cyan for β-sheet, orange for loop). **(B,C)** The conformations of LOXL2 at oxidized state and reduced state of disulfide bonds Cys351–Cys414. Black arrows show the positions of conformation changes.

For the disulfide bonds in SRCR3 domains (⑦, ⑧, and ⑨ as shown in Figure 8, Cys351–Cys414 has a high reduction potential of −269 mV, indicating that it could be a functional bond. For the other two disulfide bonds, they are predicted to have a structural role. Structurally, Cys351–Cys414 are located on the surface of the SRCR3 domain and link two loops (loop_G348-L357_, loop_D404-G421_, as shown in Figure 9A). Compared to the α-helix and β-sheet, the loop owns higher flexibility (Liljas et al., 2017), and it expands the interaction between the disulfide bond and the adjacent area. Experimentally, LOXL2 ΔSRCR3 had distinct decrease effects on the migration of ESCC cells than LOXL2 WT, but little effect on the oxidase activity of LOXL2 (Zou et al., 2020). In another study, knockout of SRCR domains 1–3 did not affect the oxidation activity of LOXL2 remarkably, which demonstrates that the SRCR1-3 domains are not indispensable for the enzyme activity (Xu et al., 2013). Therefore, the functional disulfide bond ⑦ is related to the migration and invasion of cells and should be an allosteric disulfide bond. To explore how disulfide bond ⑦ influences the conformation changes of LOXL2, we perform MD simulation with the reduced state of disulfide bond ⑦. It is shown in Figure 10A that, after cleavage of disulfide bond ⑦, the conformation of LOXL2 changes remarkably. The RMSD between oxidized and reduced states is 2.7 Å. Secondary structure changes are found on some residues. As is shown in Figures 10B,C, loop A formed by G348−V350 changes to β-sheet. This change is caused by hydrogen bond formation of the backbone of these residues, which usually act for some specific bioactive function (Liljas et al., 2017). In addition, some α-helix and β-sheet change to loops shown in Figures 10B,C, including α-helix_H416-E418 _(B), β-sheet_L458-V459 _(C), β-sheet _R598-S601 _(D), β-sheet_I621-H623 _(E), α-helix_D624-H626 _(F), β-sheet_R627-M632 _(G), β-sheet_T661-C663 _(H), β-sheet_H652-A654 _(I), and α-helix_C673-N675 _(J). These changes are also caused by hydrogen bond cleavages. Thus, disulfide bond ⑦ can affect the structures of residues which are far from the disulfide bond.

For the disulfide bonds in SRCR 4 domains (⑩, ⑪, and ⑫ as shown in Figure 8, reduction potentials of Cys464–Cys530 and Cys477–Cys543 are −215 and −270 mV, respectively (predicted to be functional role), Cys511–Cys521 is −426 mV (predicted to be the structural role). Structurally (as shown in Figure 9B), Cys464–Cys530 links two loops (loop_G461-I470_, loop_H520-G534_) which are located on the surface of SRCR4; they are characterized by high flexibility for interaction. For Cys477–Cys543, it links a α-helix (helix_I470-L480_) and a β-sheet (sheet_G540-S544_), which are two typical secondary structures with high conservation in protein and usually act for some specific function (Liljas et al., 2017). Notably, they are located on the junction of SRCR4 and the catalytic domain, which might be possible for interaction with the catalytic domain. Experimentally, the catalytic activity of LOXL2 was decreased dramatically without the fourth SRCR domain (Zou et al., 2020). On the other hand, LOXL2 ΔSRCR4 showed higher activity in ESCC cell migration and invasion than wild-type LOXL2 in ESCC cells, indicating that the functions of migration and invasion are independent of SRCR4. In addition, the SAXS and electron microscopy data revealed that SRCR4 interacts with the catalytic domain directly (Schmelzer et al., 2019). Thus, the two disulfide bonds (Cys464–Cys530 and Cys477–Cys543) are most likely to have a catalytic role.

For the disulfide bonds in catalytic domains (⑬, ⑭, ⑮, ⑯, and ⑰ in Figure 8), all of them have high reduction potential > −300 mV except ⑰ (−386 mV), indicating that they play a functional role (disulfide bonds ⑬, ⑭, ⑮, ⑯) and structural role (disulfide bond ⑰). In our previous study, LOXL2 ΔLO, which did not have the catalytic domain, had a similar activity in ESCC cell migration compared with wild-type LOXL2, indicating that the migration function of LOXL2 is independent of the catalytic domain (Zou et al., 2020). On the other hand, it can be seen from the spatial conformation that the four disulfide bonds are close to the catalytic center. Intriguingly, the reduction potentials for the five disulfide bonds in the catalytic domain are correlated with the distances between the locations of disulfide bonds and catalytic site of metal Zn (in Figure 9C), i.e., short distance (5∼18 Å) corresponds to a high reduction potential (−182∼−298 mV) which corresponds to a functional role.

In Figure 9C, based on structure alignment, the β-sheet structures in the catalytic domain are aligned with the β-galactosidase type; those aligned commendably apart are defined as structure core (colored yellow), and the nonoverlapping regions are defined as extra segments (ES1–3, colored orange) (Zhang et al., 2018). In the core area, the catalytic site is surrounded by 4 pairs of disulfide bonds ⑬, ⑭, ⑮, and ⑯ in different extents of distances. Moreover, disulfide bond ⑰ links the edge of the core area and ES3. ES1 (residues 568–580) contains a α-helix, which links to the core through disulfide bond ⑭. The major structure element of ES2 (residues 614–634) is a β-hairpin, which accommodates a highly conserved copper-binding motif. ES2 is linked to ES1 through disulfide bond ⑬, which may act its stabilization function to this crucial β-hairpin. ES3 (residues 745–774) is at the C terminus of the catalytic domain and is responsible for the interaction with the SRCR3 domain. In a whole view, beside structural characters, disulfide bonds ⑬, ⑭, ⑮, and ⑯ are preferred to have a catalytic role, while ⑰ has a structural role.

In summary, seven disulfide bonds are predicted to have a functional role in LOXL2. The disulfide bond of Cys351–Cys414 in SRCR3 is most likely to act its allosteric function. In the catalytic domain, four disulfide bonds play a role in catalytic function. The remaining two catalytic function bonds are located in the SRCR4 domain, which is close to the catalytic domain.

### The Performance of Work Distributions in Non-Equilibrium Free Energy Calculations

**FIGURE 11:**
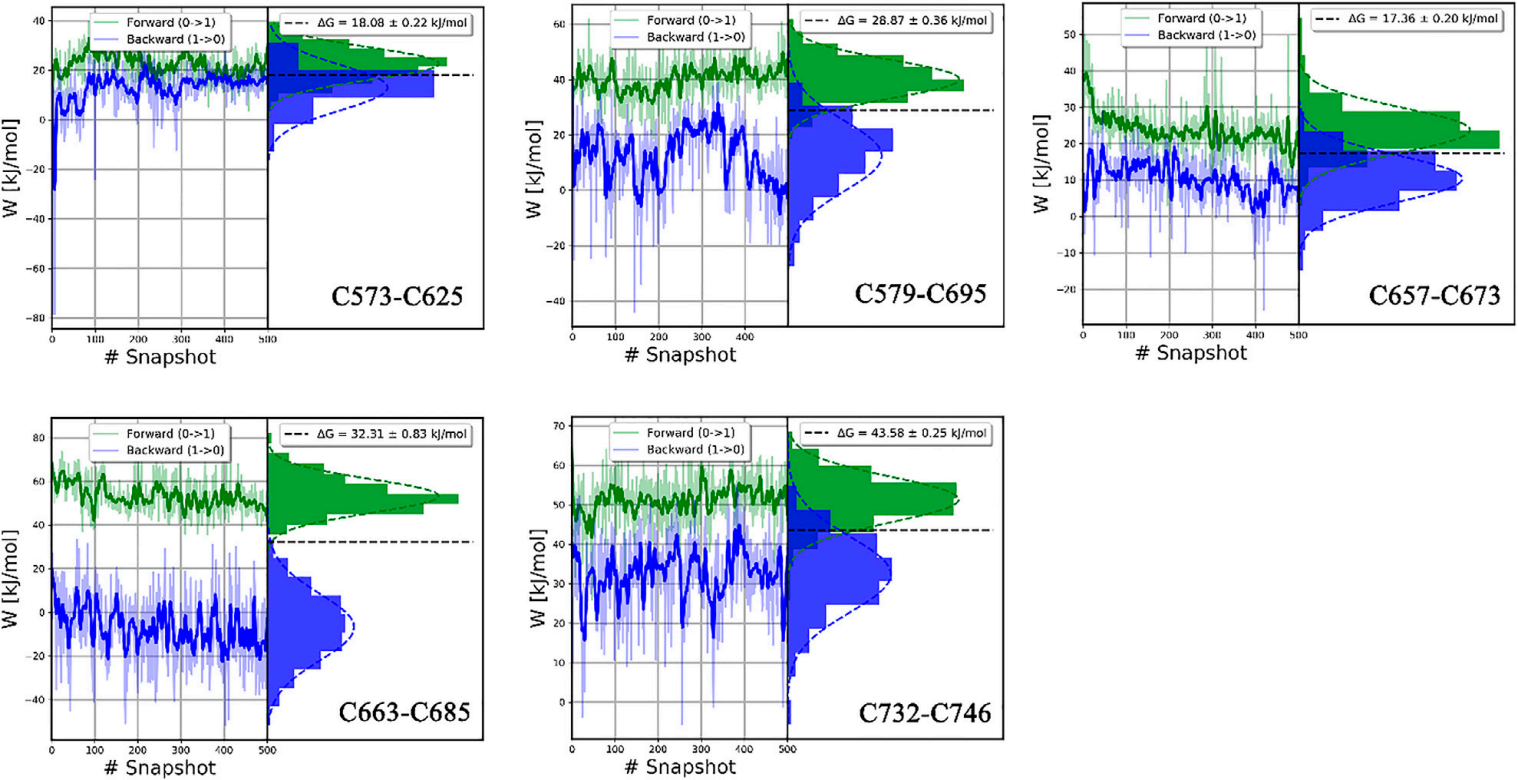
The histograms of the forward and backward work for each disulfide bond in the catalytic domain. Plotted by the *pmx* program.

In this study, all the work values for cleavage of disulfide bonds were calculated by the *pmx* program (Gapsys et al., 2015), as shown in Figure 11; Supplementary Figure S3. The δHλ/δλ curve as a function of λ in typical forward and backward transformations is shown in Supplementary Figure S2, indicating the high convergence of transformation. Independent work values for 500 snapshots of forward and backward transitions are generated, and the total free energy difference ΔG for each disulfide bond is shown on the top right of each picture.

In our study, ΔG values of disulfide bonds in LOXL2 were calculated by the BAR estimator in the *pmx* program. The accuracy of results can be assessed in various ways. One indicator of accuracy is the convergence of systematic dynamics of work value (Jarzynski, 2006). In Figure 11 and Supplementary Figure S3, the work values do not have a large drift, especially in the forward distribution. In addition, the distributions of work values of 500 snapshots for all disulfide bonds are concentrated and can be fitted to Gaussian distribution. Second, the overlap of the forward and backward work distribution is important to the binding free energy (Mey et al., 2020). The accuracy of the CGI and BAR estimator is sensitive to the overlap. We can see an apparent overlap for each disulfide bond as shown in Figure 11 and Supplementary Figure S3. In our study, 500 replicas of non-equilibrium simulations are performed to generate a free energy difference. Third, the standard errors of ΔG are less than 1.4 kJ/mol in all our calculations, indicating that our results are stable (Shirts et al., 2003). Thus, all these confirm that the ΔGs are reliable.

## Conclusion

Disulfide bonds exist in many proteins and play important roles in the structure and function of proteins (Pijning and Hogg, 2019). In LOXL2, seventeen disulfide bonds are formed. The functional bonds can be divided into two types: catalytic and allosteric functions. The allosteric function of the disulfide bond in LOXL2 corresponds to the ability of metastasis and invasion of cancer cells, and the catalytic function corresponds to the enzyme activity. In this paper, we determine the role of each disulfide bond by reduction potential calculation with nonequilibrium alchemical simulations. Seven disulfide bonds are predicted to have a functional role, whereas others have a structural role. One of the functional disulfide bonds (Cys351–Cys414) is the allosteric bond in SRCR3. Two catalytic functions of disulfide bonds (Cys464–Cys530 and Cys477–Cys543) are found in the SRCR4 domain, which is close to the catalytic domain. In the catalytic domain, four disulfide bonds are predicted to be of catalytic function, i.e., Cys573–Cys625, Cys579–Cys695, Cys657–Cys673, and Cys663–Cys685. Notably, the reduction potentials for the five disulfide bonds in the catalytic domain are correlated with the distances between the location of disulfide bonds and catalytic sites, i.e., short distance leads to high reduction potential. In addition, no functional disulfide bond is found in the SRCR 1 and SRCR2 domains.

## Data Availability

The original contributions presented in the study are included in the article/Supplementary Material; further inquiries can be directed to the corresponding authors.
