# Supplementary material for: Redox Potentials of Disulfide Bonds in LOXL2 Studied by Nonequilibrium Alchemical Simulation
Source: Front Chem. 2021 Dec 14;9:797036. doi: 10.3389/fchem.2021.797036 (PMC8713139; doi:10.3389/fchem.2021.797036)
Supplement: Supplementary file 1 [file DataSheet1.PDF]

Supporting Information

Redox Potentials of Disulfide Bonds in LOXL2 Studied by Nonequilibrium Alchemical Simulation

Lirui Lin[1,2], Haiying Zou[1,3], Wenjin, Li[4], Li-Yan Xu[*3,5], En-Min Li[*1,3], Geng Dong[*1,2]

[1] Department of Biochemistry and Molecular Biology, Shantou University Medical College, Shantou, 515041, PR China

[2] Medical Informatics Research Center, Shantou University Medical College, Shantou, 515041, PR China

[3] Key Laboratory of Molecular Biology in High Cancer Incidence Coastal Area of Guangdong Higher Education Institutes, Shantou University Medical College, Shantou, 515041, PR China

[4]Institute for Advanced Study, Shenzhen University, Shenzhen 518060, China

[5] Cancer Research Center, Shantou University Medical College, Shantou, 515041, PR China

Correspondence to lyxu@stu.edu.cn,
or En-Min Li, E-mail: nmli@stu.edu.cn,
or Geng Dong, E-mail: gdong@stu.edu.cn

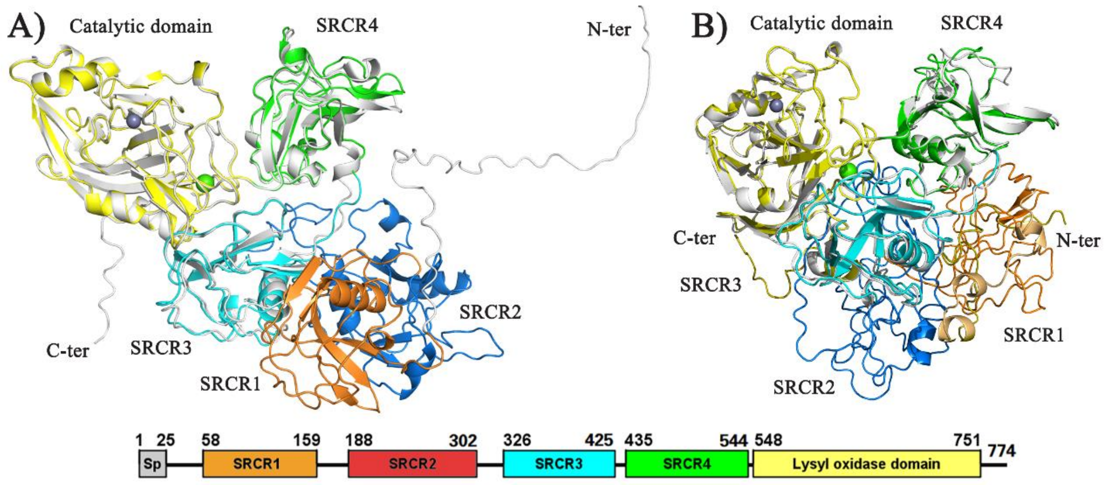

FIGURE S1 Structure alignments of LOXL2 between crystal and predicted structures. A) The missing domain SRCR1-2 of LOXL2 was predicted by AlphaFold2, alignment RMSD = 0.62 Å (Jumper et al., 2021; Bershtein, Kleiner, and Mishmar, 2021); B) Predicted by I-TASSA, RMSD = 0.75 Å (Roy, Kucukural, and Zhang, 2010). Crystal structure PDB ID: 5ZE3, Sp: signal peptide. Bound zinc and calcium are displayed as spheres.

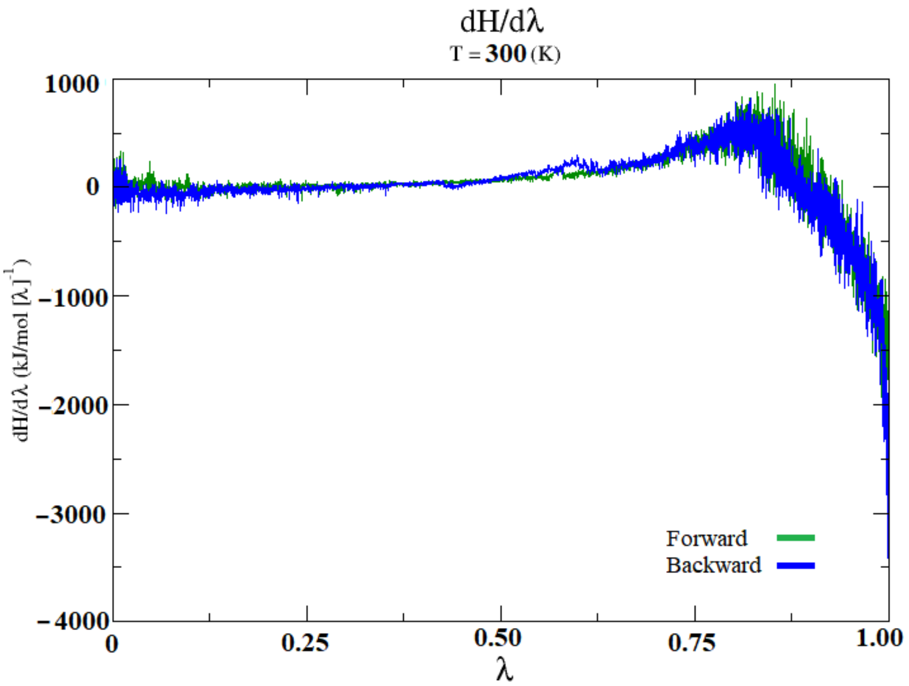

FIGURE S2 An example of the $\delta H_\lambda/\delta_\lambda$ results at different λ in the forward (green) and backward (blue) transformations.

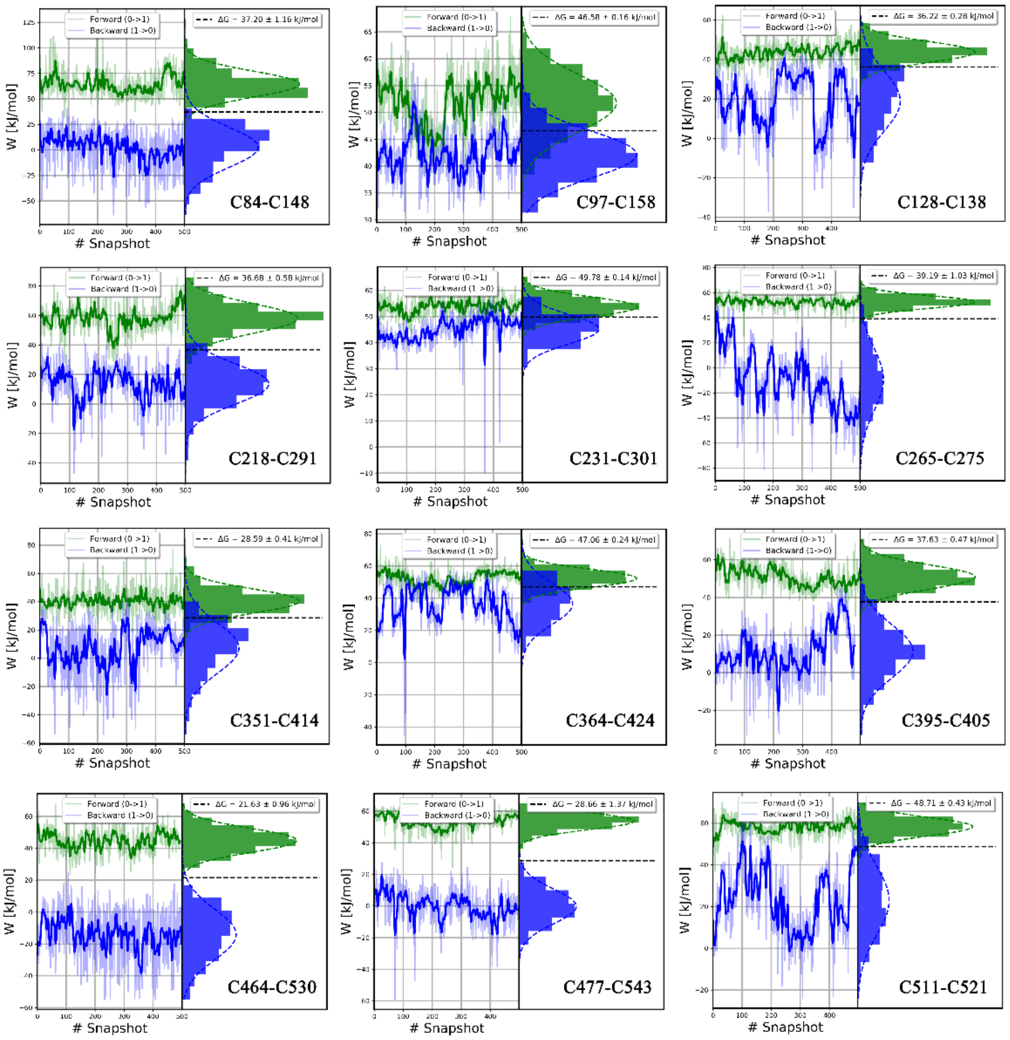

FIGURE S3 The histograms of the forward and backward work for each disulfide bond in SRCR1-4 domain. Plotted by *pmx* program (Gapsys et al., 2015).

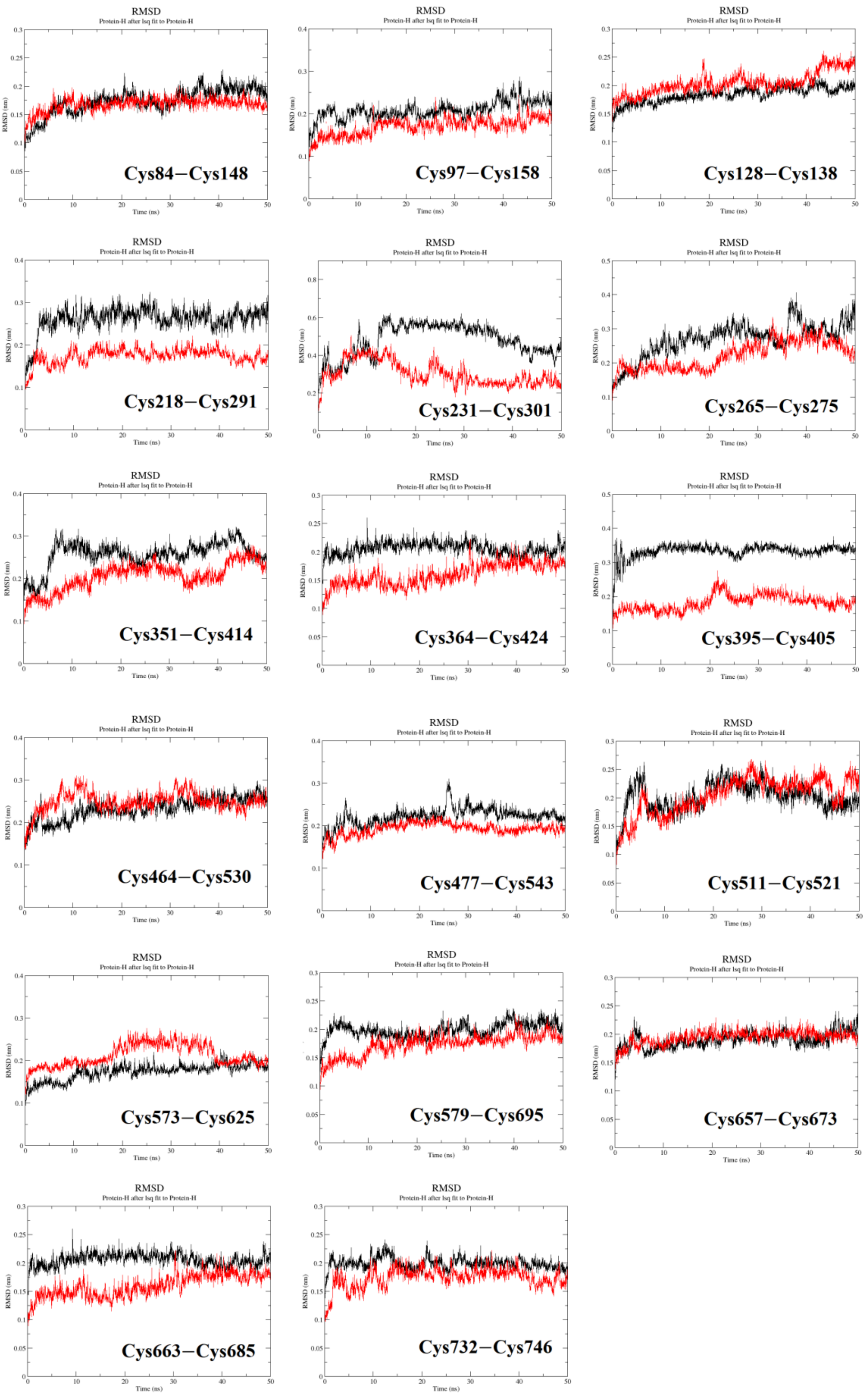

FIGURE S4 The RMSDs for LOXL2 in oxidized and reduced states of disulfide bonds

Parameter for Hybrid Cysteine/Cystine Force Field

Crooks Gaussian intersection (CGI) method are used to calculate the difference in free energy and the redox potential of proteins undergoing reduction of their disulfide bonds, as shown in Figure 4. In CGI, the parameter λ switches the system in an alchemical transformation from the oxidation to reduction state. Thereby, the disulfide bond needs to be opened through such a transformation. When a bond shall be opened, the bonded interactions (not only the bond itself but also the angles and dihedrals that enclose the bond) shall be transformed to nonbonded interactions. This represents a challenge in the setup of the free-energy calculations, as it requires the introduction of nonbonded interactions between atoms, which in the oxidized state were bonded, entailing an exclusion of any nonbonded term in this state.

Here, we constructed a hybrid cysteine/cystine force field on the basis of the CHARMM27 force field to enable the transformation between cystine and two free cysteines in the MD software GROMACS. This single topology approach was chosen as it can be expected to converge faster than a dual topology approach, as it minimizes the number of dummy atoms and perturbed degrees of freedom. We introduced a new cysteine residue type, which extends the disulfide bonded cysteine (CYS2) in the CHARMM27 force field by adding one atom HUD and two virtual sites (V_c and V_s). The dummy atom and virtual sites do not have bonded interactions and LJ interactions. The new residue was named CYD.

HUD with a mass of a hydrogen atom is bonded to the sulfur. The distance between V_c (V_s) and CA (CB) is constrained to be the same as the one between CB (S) and CA (CB). Thus, the virtual sites Vc and Vs colocalize with CB and S, respectively. The nonbonded interactions between V_c (V_s) and those atoms in CYD separated by no more than three bonds with CB (S) are excluded. At $\lambda = 0$, the dummy HUD and two virtual sites (V_c and V_s) have zero charge and thus have no interactions with any other atoms in the system. Therefore, CYD at $\lambda = 0$ corresponds exactly to CYS2.

Analogously, we want to reproduce the open cysteine state, CYS, in CHARMM27 with CYD at $\lambda = 1$. At $\lambda = 1$, HUD is transformed into a hydrogen atom and shows all bonded interactions of the hydrogen atom bonded to the sulfur in CYS, but several nonbonded interactions with HUD, as listed in Table 1, are missing. There are some bonded interactions present in cystine which ought to be absent in two independent cysteine residues, the most obvious being the bond S1−S2 and the angle CB1−S1−S2. Here, the subscripts 1 and 2 refer to atoms in CYD1 and CYD2, respectively.

These bond and angle potentials can be switched to zero at $\lambda = 1$, they will lead to an exclusion of some of the nonbonded interactions necessary to represent the two CYS. To circumvent this exclusion, V_c and V_s carry nonbonded interactions at $\lambda = 1$. In addition, some interactions, such as the LJ interaction between CA1 and S2, are 1−4 interactions at λ=0 but short-range LJ interactions at λ=1. The necessary transformation of these nonbonded interactions are added to the pair list and are summarized in Table S1.

Table S1. Nonbonded Interactions Added to the Pair List To Enable CYD at λ = 0 and λ = 1 to Reproduce CYS2 and CYS, Respectively[a]

**Particle 1**	**Particle 2**	**λ=0**	**λ=1**
S1	CA_2	1-4	short
S2	CA_1	1-4	short
S_1[b]	$HB1_2$	1-4	short
S2	$HB1_1$	1-4	short
CB_1[b]	CB_2	1-4	short
V_{s1}	CB_2	off	on
V_{s2}	CB_1	off	on
V_{s1}	S_2	off	on
HUD_1	CB_2	off	on
HUD_2	CB_1	off	on
HUD_1	S_2	off	on
HUD_2	S_1	off	on
HUD_1	HUD_2	off	on
V_c[c]	HN	off	off
V_s[c]	N	off	off
V_c[c]	O	off	off
V_s[c]	HA	off	off
V_s[c]	C	off	off

[a] GROMACS distinguishes nonbonded interactions between atoms separated by exactly three bonds, i.e., 1−4 interactions, and short-range interactions between atoms separated by more than three bonds. Here, "1−4" and "short" refer to the former and latter, respectively.

[b] This applies to the two HB1s. [c] This applies to both CYDs. The subscripts 1 and 2 refer to atoms in CYD1 and CYD2, respectively.

Reference

Bershtein, S., Kleiner, D., and Mishmar, D. (2021). Predicting 3D protein structures in light of evolution, *Nat Ecol Evol*

Gapsys, V., Michielssens, S., Seeliger, D., and de Groot, B. L. (2015). pmx: Automated protein structure and topology generation for alchemical perturbations, *J Comput Chem* 36: 348-54

Jumper, J., Evans, R., Pritzel, A., Green, T., Figurnov, M., Ronneberger, O., Tunyasuvunakool, K., Bates, R., Zidek, A., Potapenko, A., Bridgland, A., Meyer, C., Kohl, S. A. A., Ballard, A. J., Cowie, A., Romera-Paredes, B., Nikolov, S., Jain, R., Adler, J., Back, T., Petersen, S., Reiman, D., Clancy, E., Zielinski, M., Steinegger, M., Pacholska, M., Berghammer, T., Bodenstein, S., Silver, D., Vinyals, O., Senior, A. W., Kavukcuoglu, K., Kohli, P., and Hassabis, D. (2021). Highly accurate protein structure prediction with AlphaFold, *Nature*

Roy, A., Kucukural, A., and Zhang, Y. (2010). I-TASSER: a unified platform for automated protein structure and function prediction, *Nat Protoc* 5: 725-38
