# Supplementary figures and images for: Redox Potentials of Disulfide Bonds in LOXL2 Studied by Nonequilibrium Alchemical Simulation
Source: Front Chem. 2021 Dec 14;9:797036. doi: 10.3389/fchem.2021.797036 (PMC8713139; doi:10.3389/fchem.2021.797036)

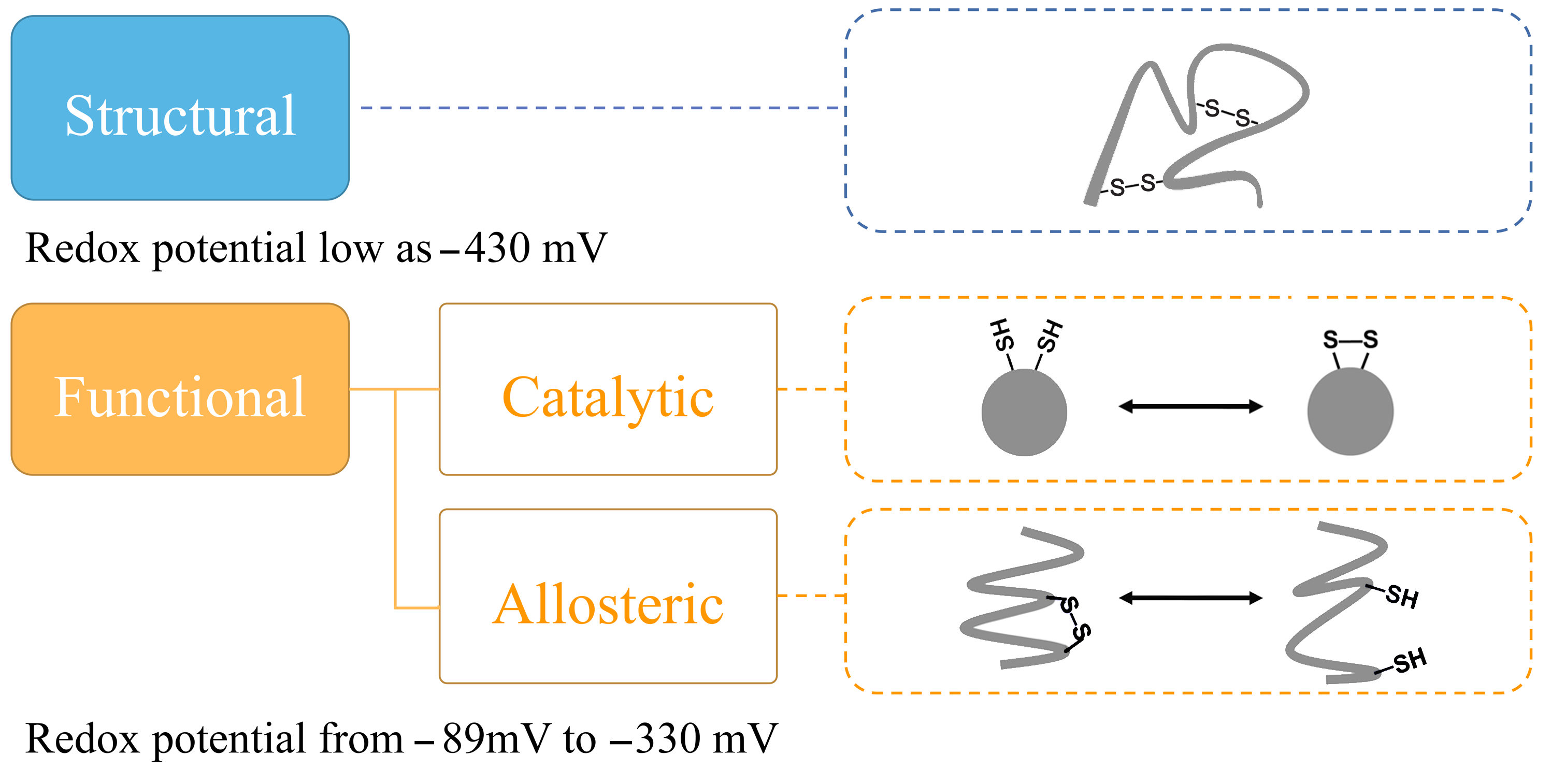

Supplement: Supplementary file 2 [file DataSheet2.ZIP › Raw Date for Redox potential LOXL2-797036/Figure 2/Classification of disulfide bond.png]

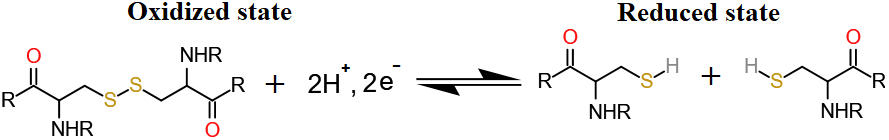

Supplement: Supplementary file 2 [file DataSheet2.ZIP › Raw Date for Redox potential LOXL2-797036/Figure 4/Redox reaction.png]

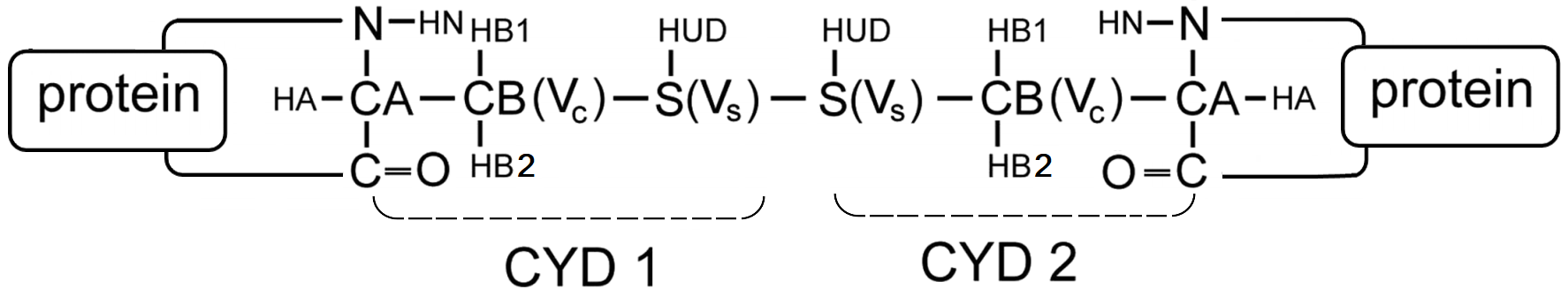

Supplement: Supplementary file 2 [file DataSheet2.ZIP › Raw Date for Redox potential LOXL2-797036/Figure 5/The scheme of CYD topology.png]

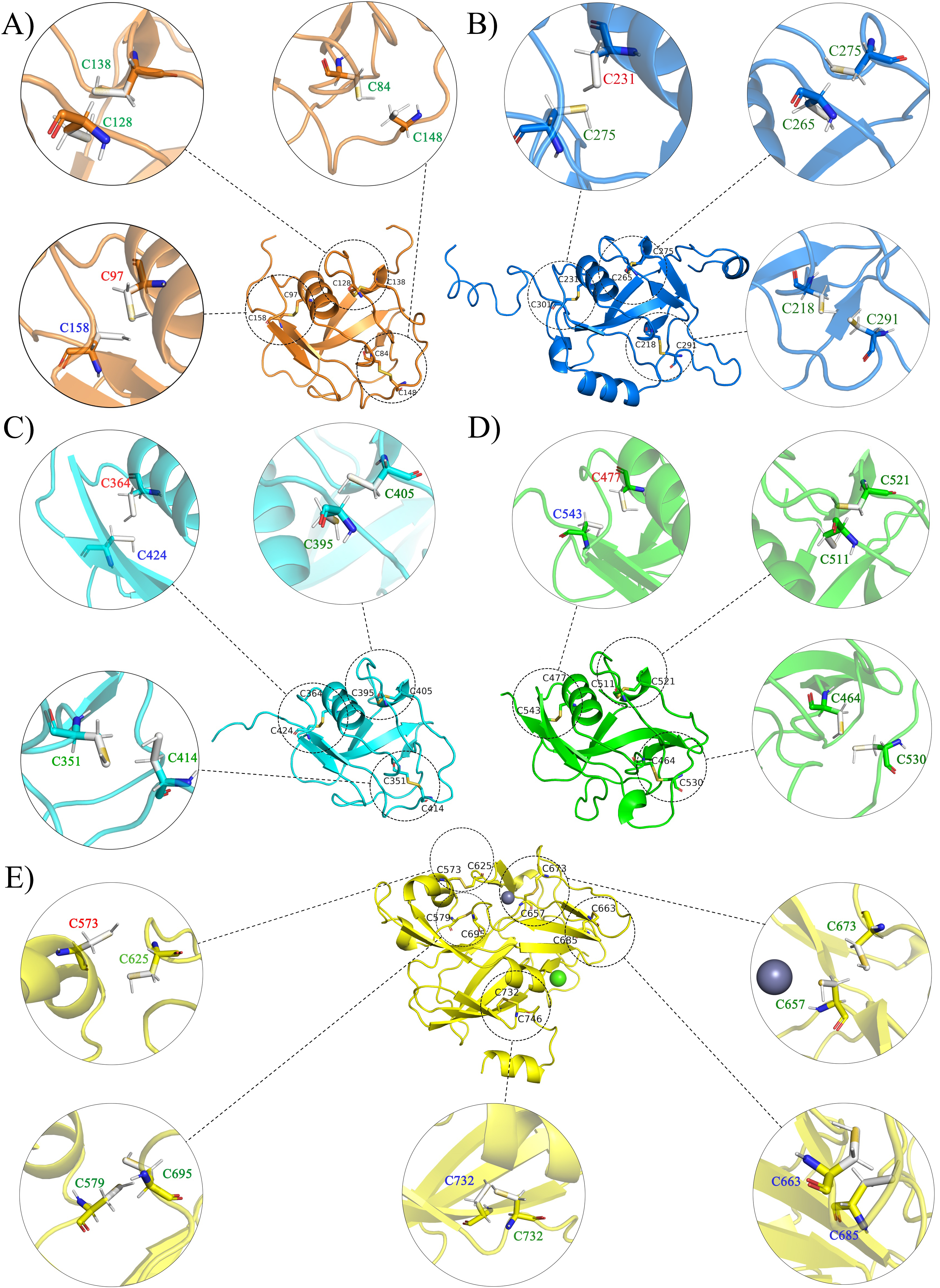

Supplement: Supplementary file 2 [file DataSheet2.ZIP › Raw Date for Redox potential LOXL2-797036/Figure 6/The oxidized and reduced states of each disulfide bond.png]

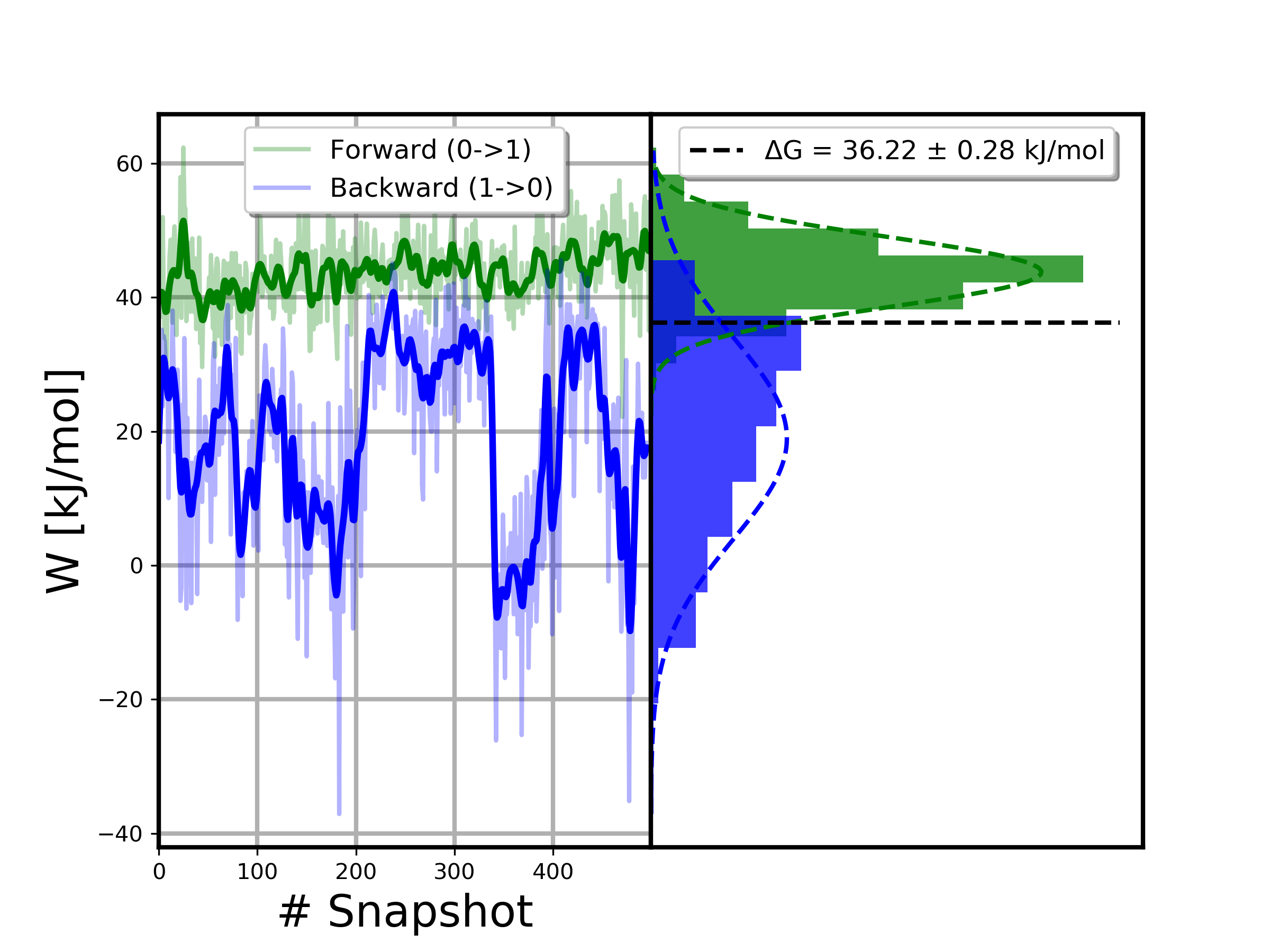

Supplement: Supplementary file 2 [file DataSheet2.ZIP › Raw Date for Redox potential LOXL2-797036/Figure 8 & 10 & Table1/wplot_1SRCR-C128-C138.png]

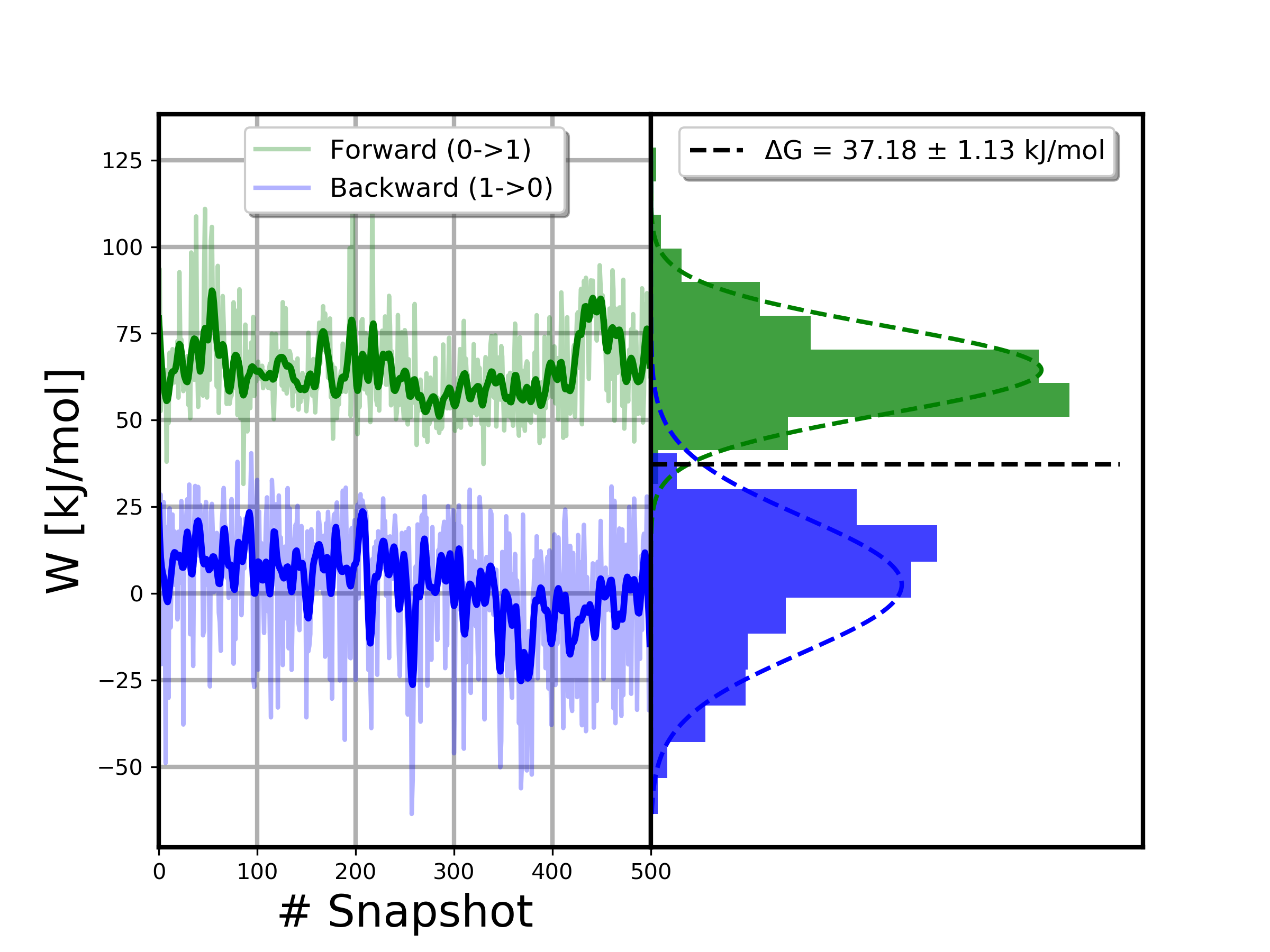

Supplement: Supplementary file 2 [file DataSheet2.ZIP › Raw Date for Redox potential LOXL2-797036/Figure 8 & 10 & Table1/wplot_1SRCR-C84-C148.png]

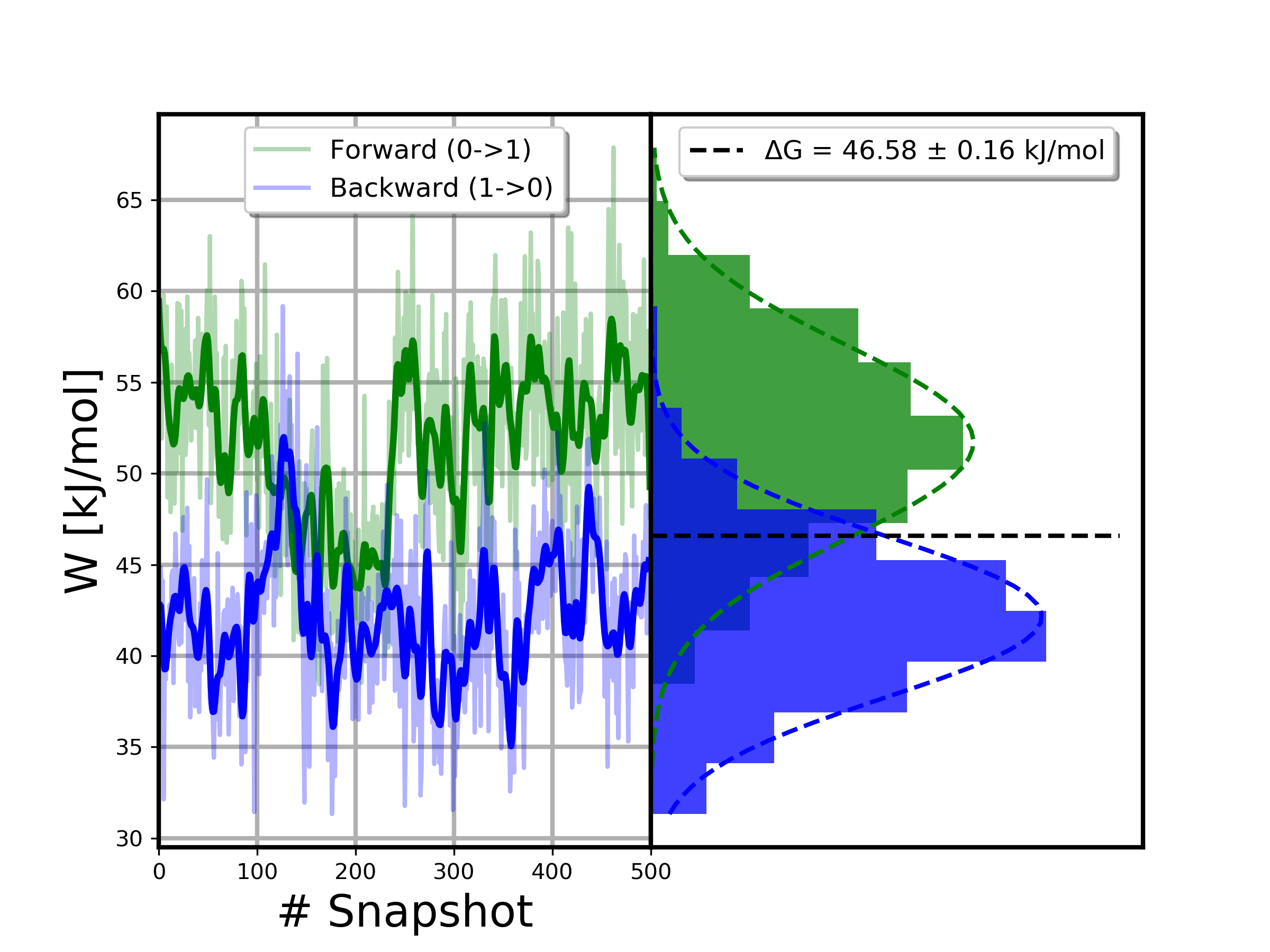

Supplement: Supplementary file 2 [file DataSheet2.ZIP › Raw Date for Redox potential LOXL2-797036/Figure 8 & 10 & Table1/wplot_1SRCR-C97-C158.png]

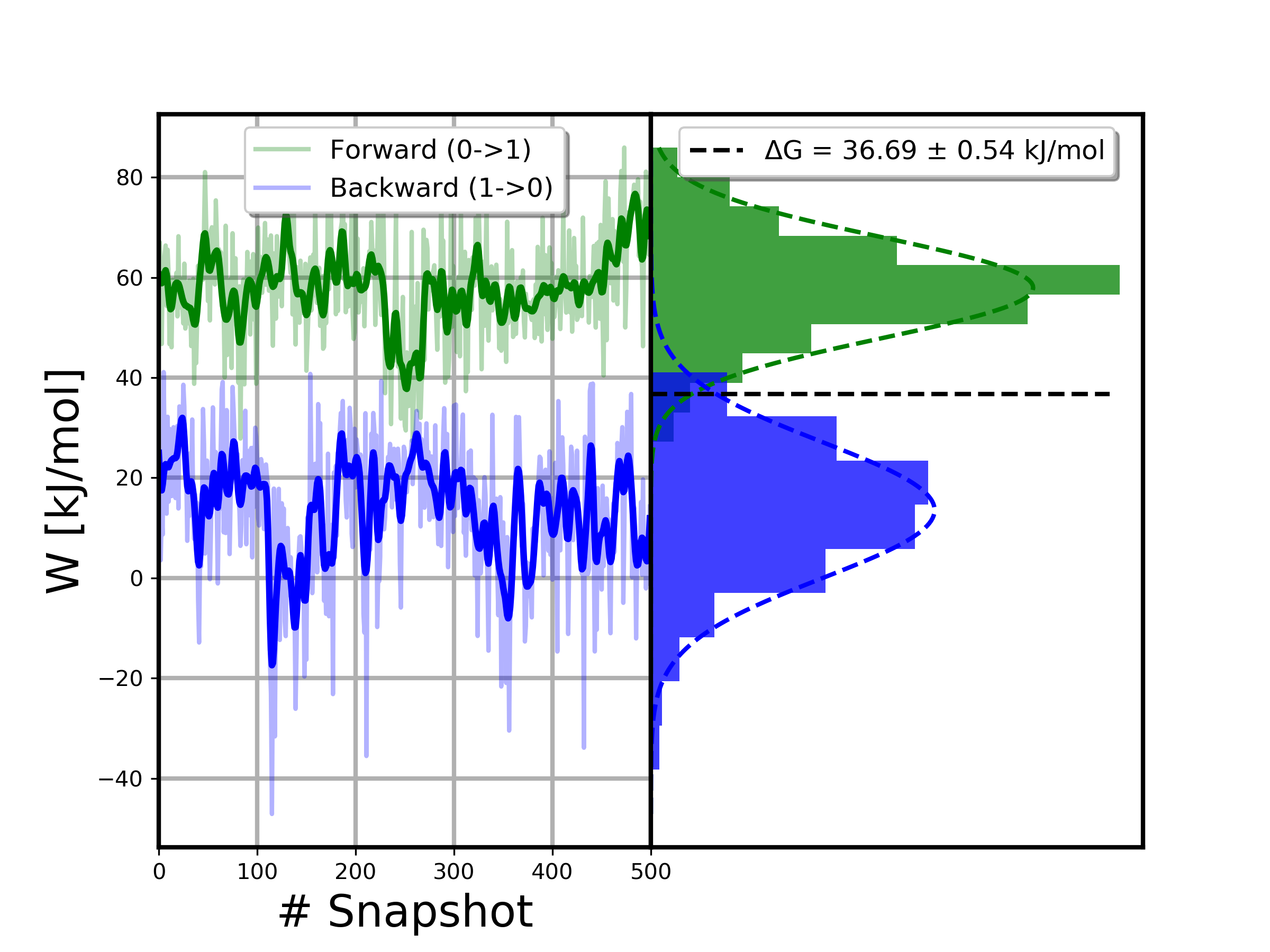

Supplement: Supplementary file 2 [file DataSheet2.ZIP › Raw Date for Redox potential LOXL2-797036/Figure 8 & 10 & Table1/wplot_2SRCR-C218-C291.png]

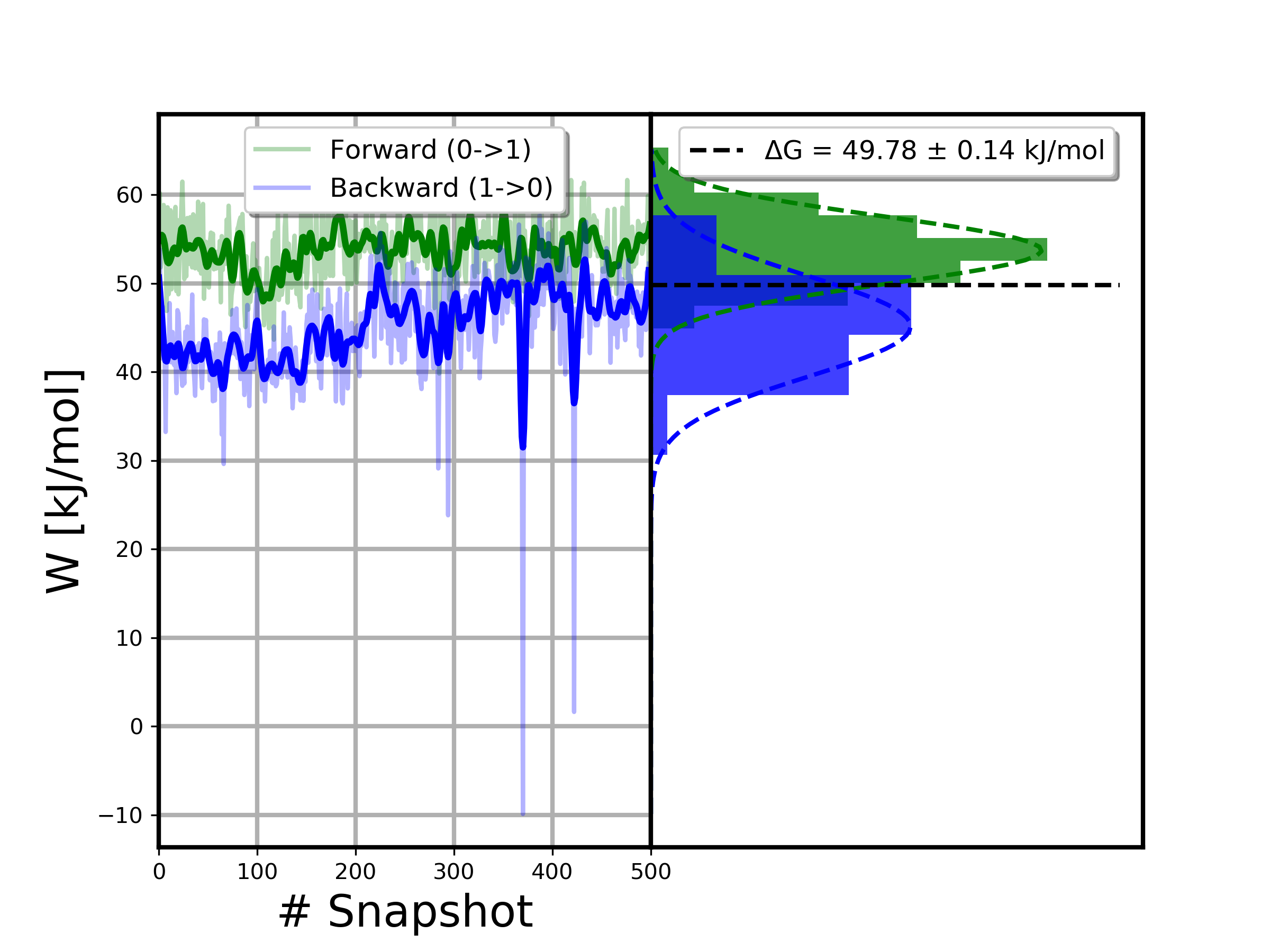

Supplement: Supplementary file 2 [file DataSheet2.ZIP › Raw Date for Redox potential LOXL2-797036/Figure 8 & 10 & Table1/wplot_2SRCR-C231-C301.png]

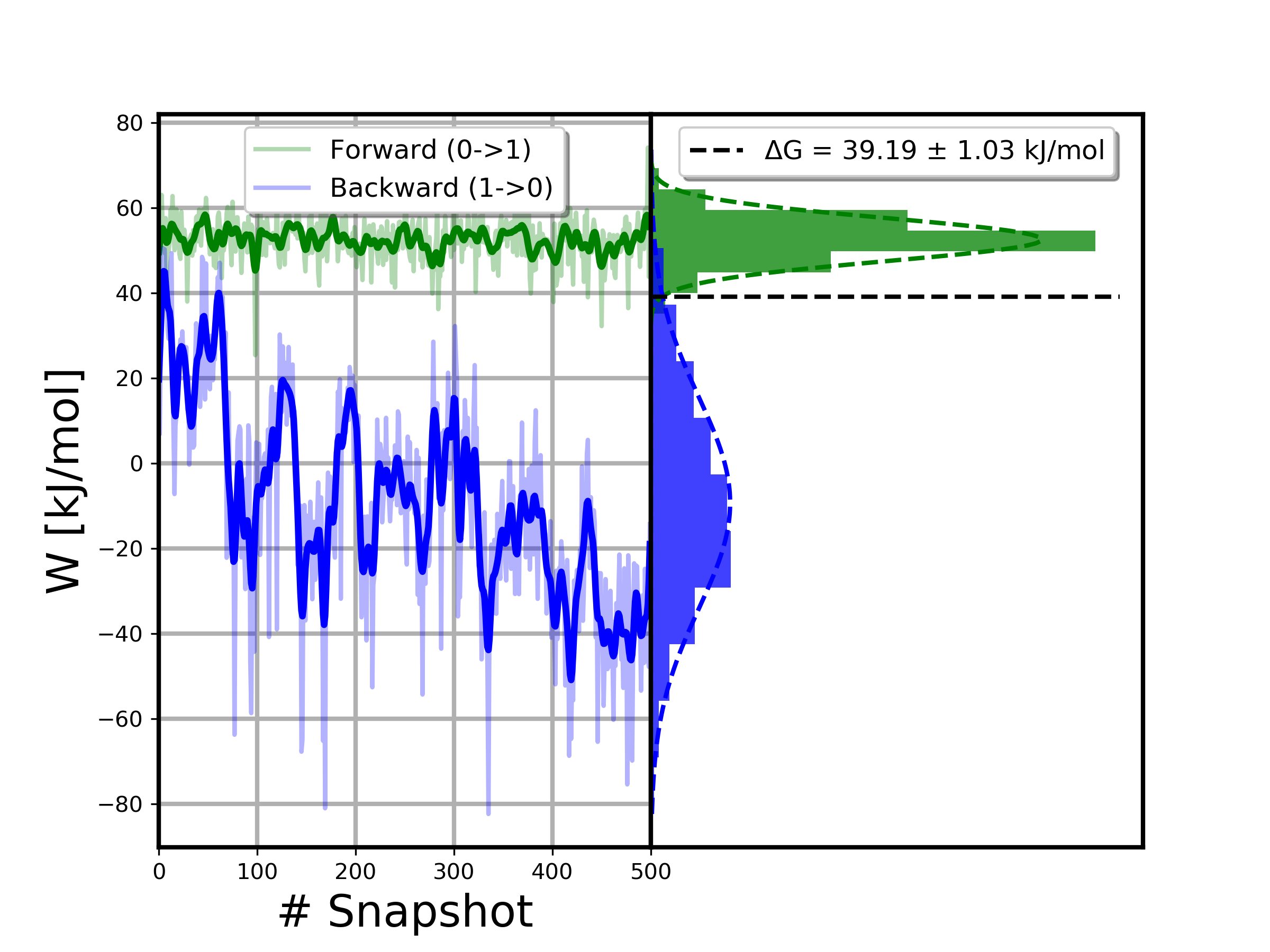

Supplement: Supplementary file 2 [file DataSheet2.ZIP › Raw Date for Redox potential LOXL2-797036/Figure 8 & 10 & Table1/wplot_2SRCR-C265-C275.png]

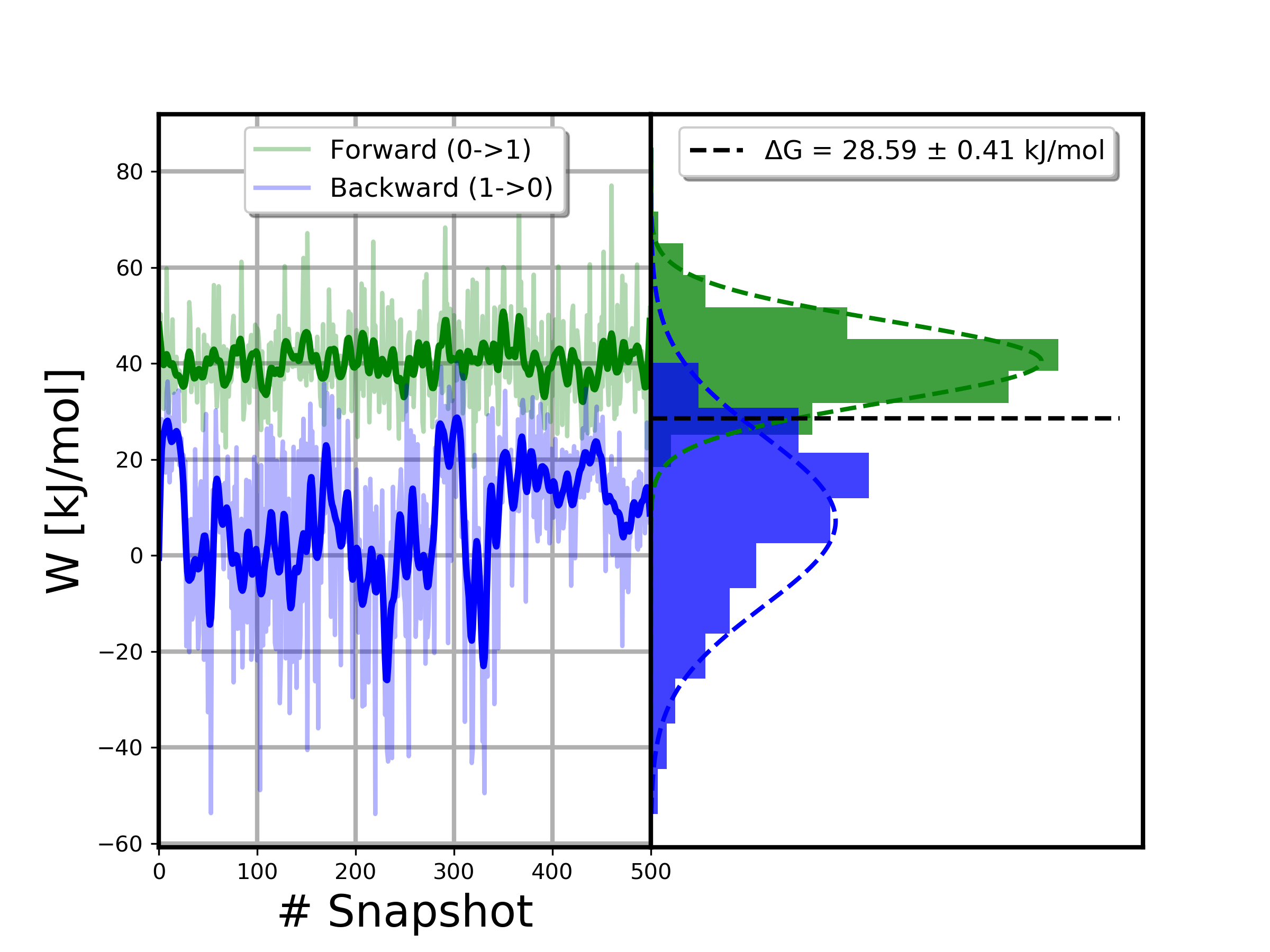

Supplement: Supplementary file 2 [file DataSheet2.ZIP › Raw Date for Redox potential LOXL2-797036/Figure 8 & 10 & Table1/wplot_3SRCR-C351-C414.png]

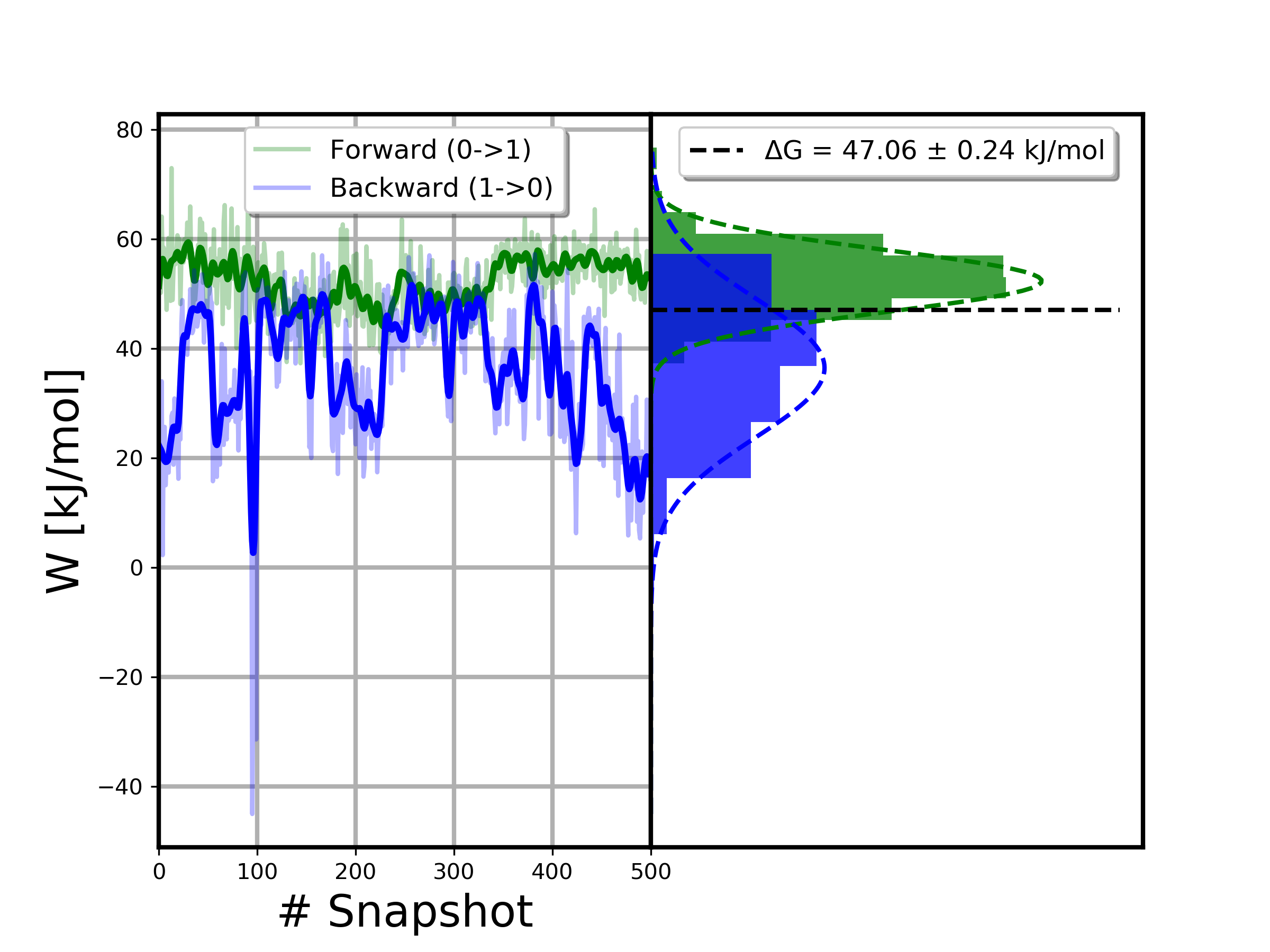

Supplement: Supplementary file 2 [file DataSheet2.ZIP › Raw Date for Redox potential LOXL2-797036/Figure 8 & 10 & Table1/wplot_3SRCR-C364-C424.png]

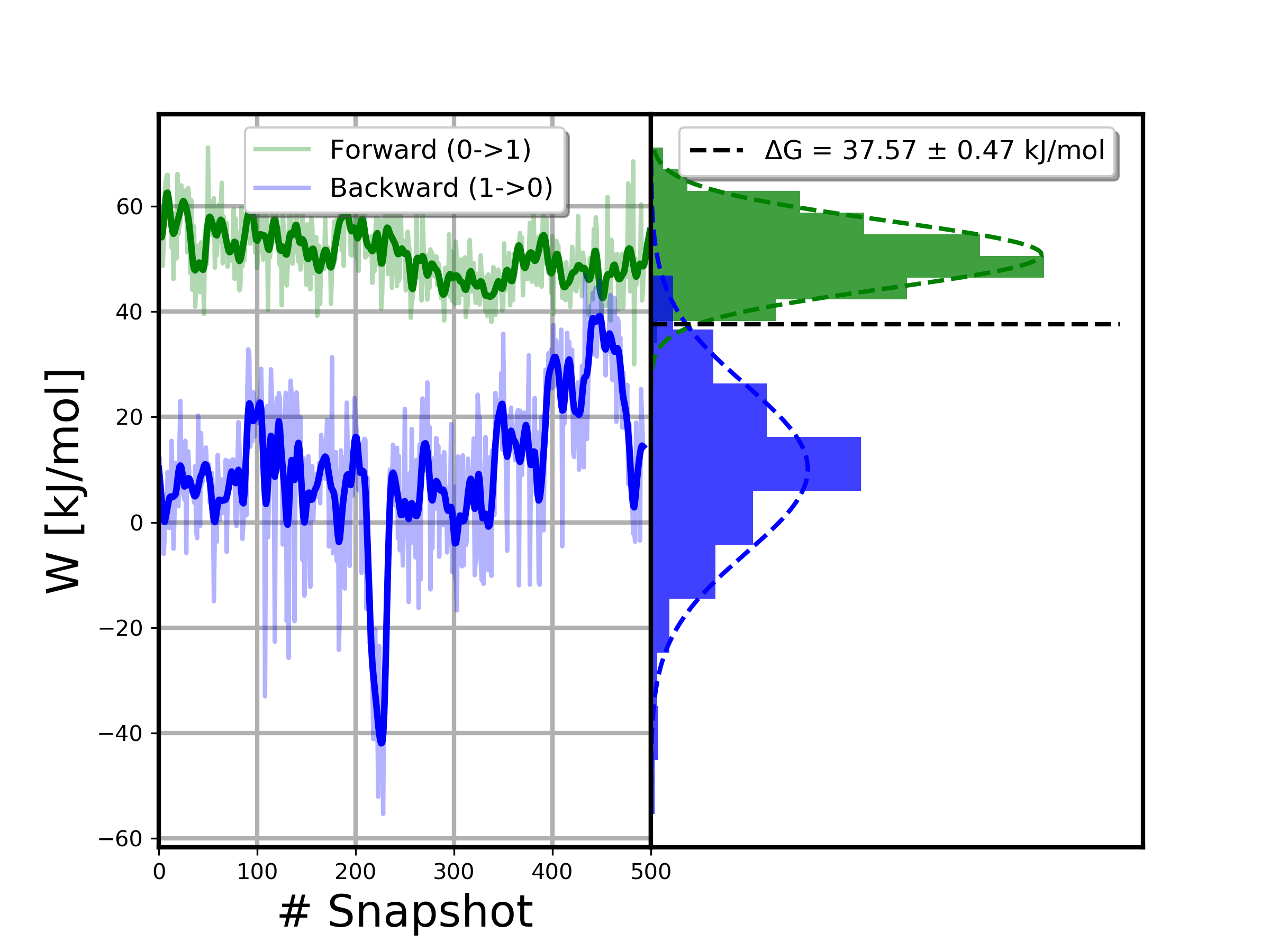

Supplement: Supplementary file 2 [file DataSheet2.ZIP › Raw Date for Redox potential LOXL2-797036/Figure 8 & 10 & Table1/wplot_3SRCR-C395-C405.png]

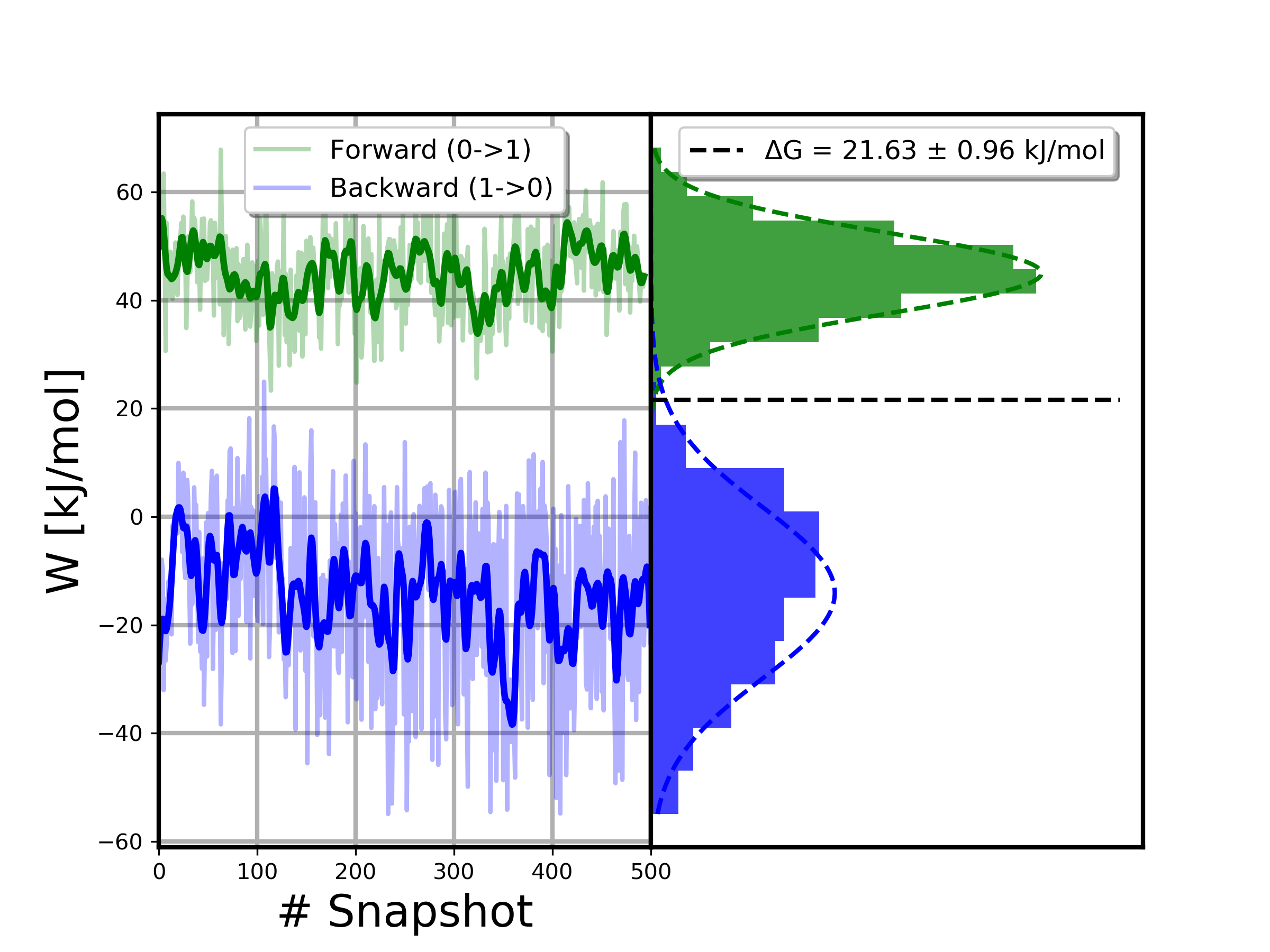

Supplement: Supplementary file 2 [file DataSheet2.ZIP › Raw Date for Redox potential LOXL2-797036/Figure 8 & 10 & Table1/wplot_4SRCR-C464-C530.png]

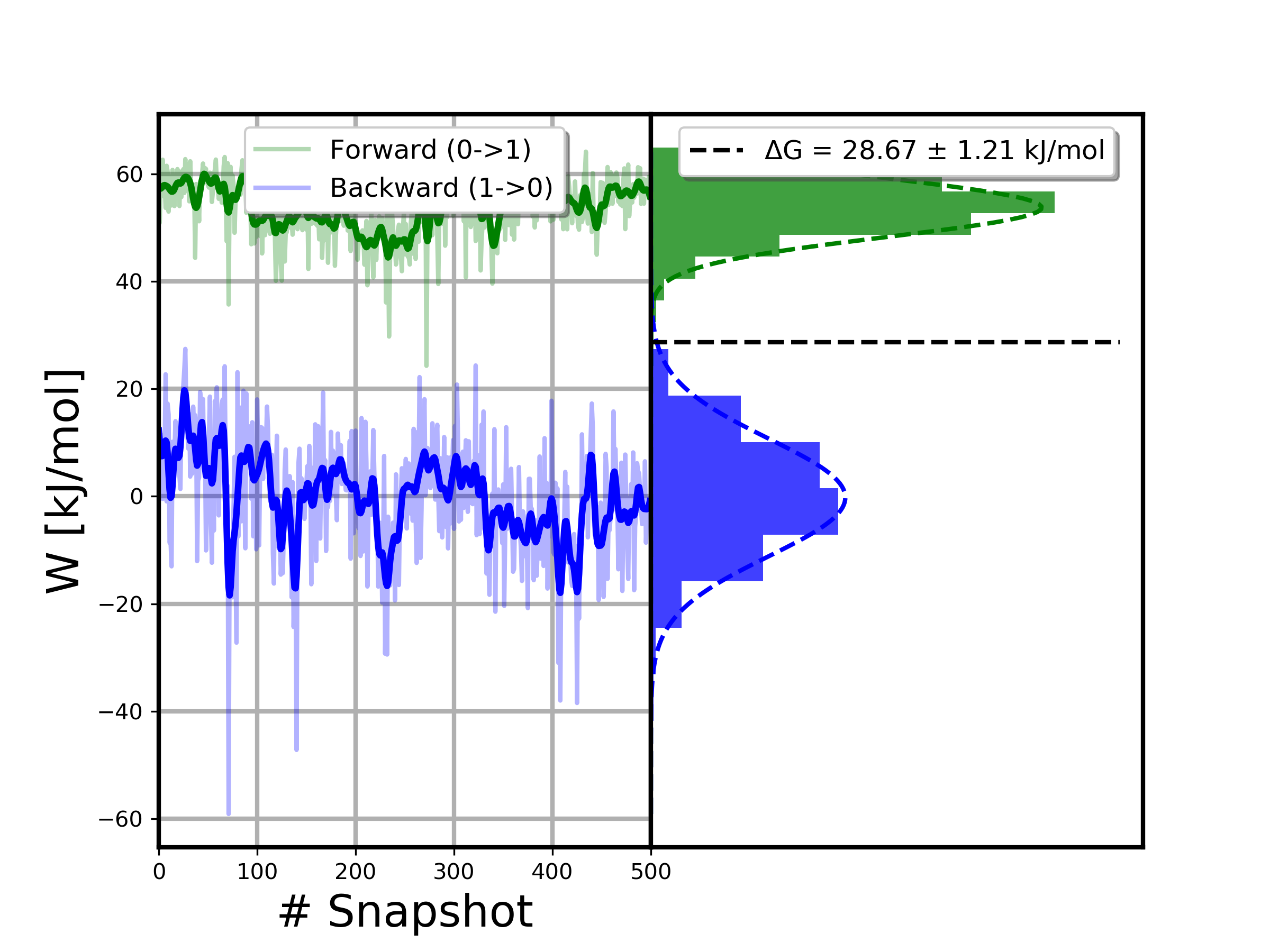

Supplement: Supplementary file 2 [file DataSheet2.ZIP › Raw Date for Redox potential LOXL2-797036/Figure 8 & 10 & Table1/wplot_4SRCR-C477-C543.png]

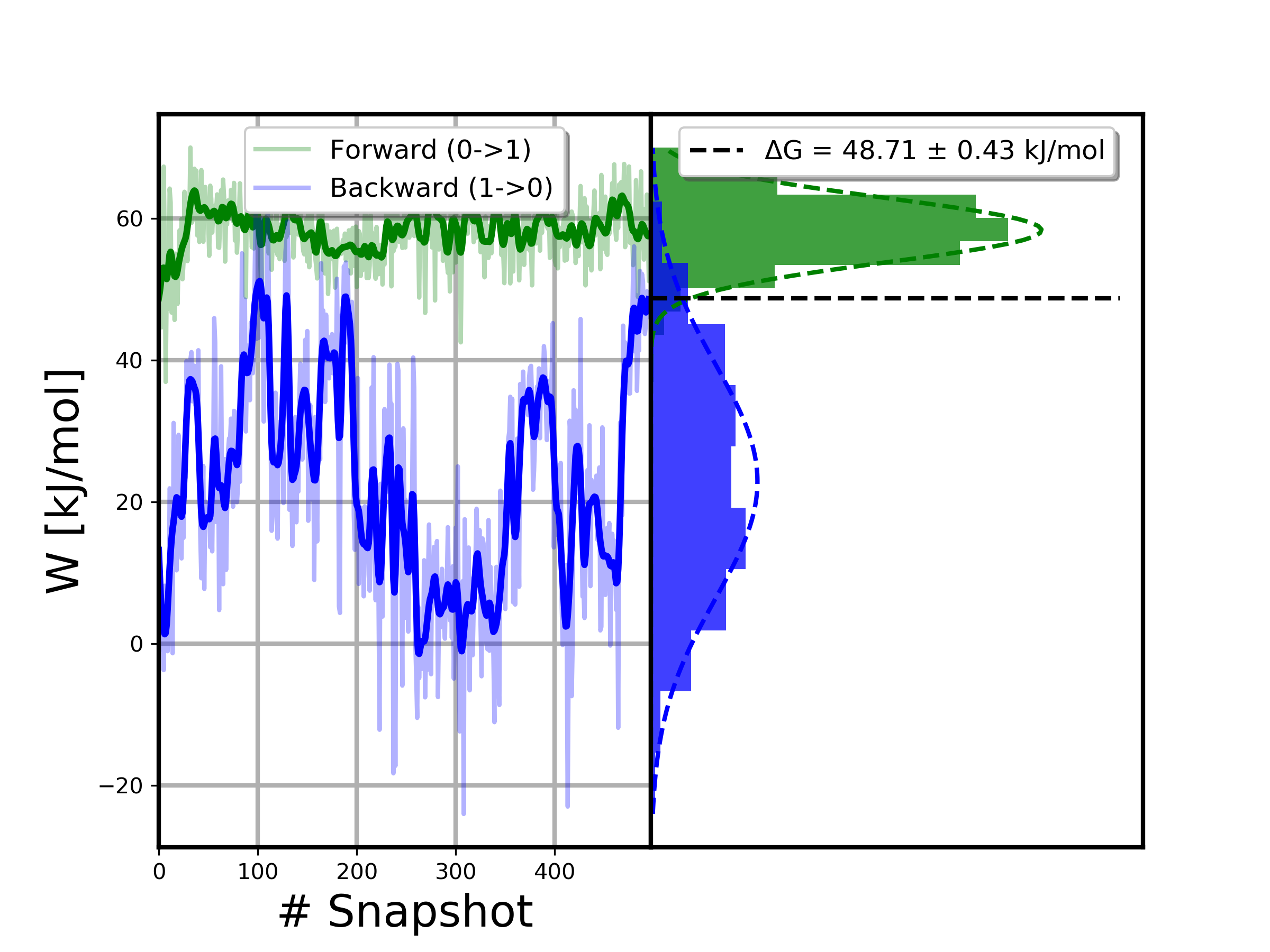

Supplement: Supplementary file 2 [file DataSheet2.ZIP › Raw Date for Redox potential LOXL2-797036/Figure 8 & 10 & Table1/wplot_4SRCR-C511-C521.png]

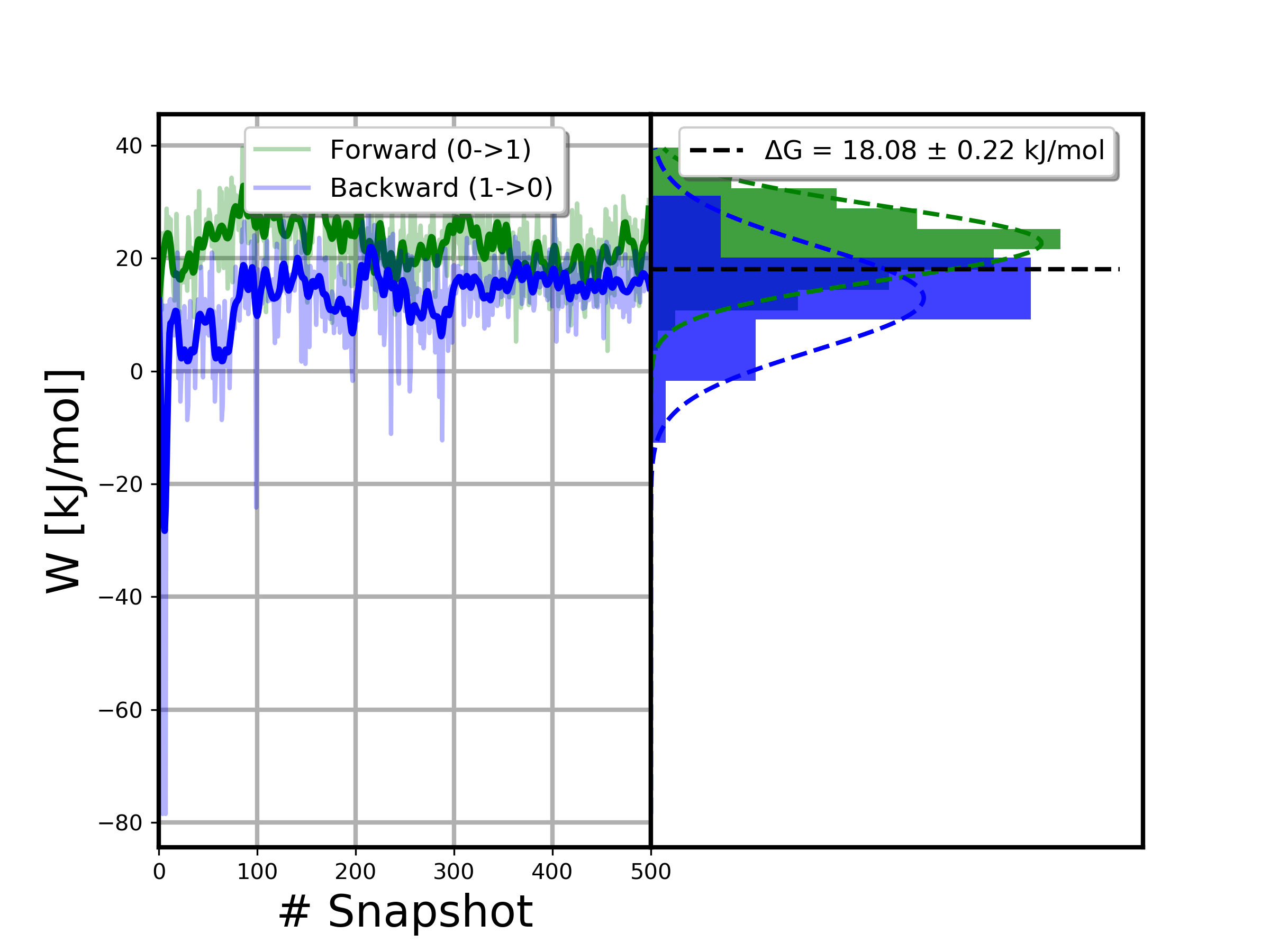

Supplement: Supplementary file 2 [file DataSheet2.ZIP › Raw Date for Redox potential LOXL2-797036/Figure 8 & 10 & Table1/wplot_catalytic-C573-C625.png]

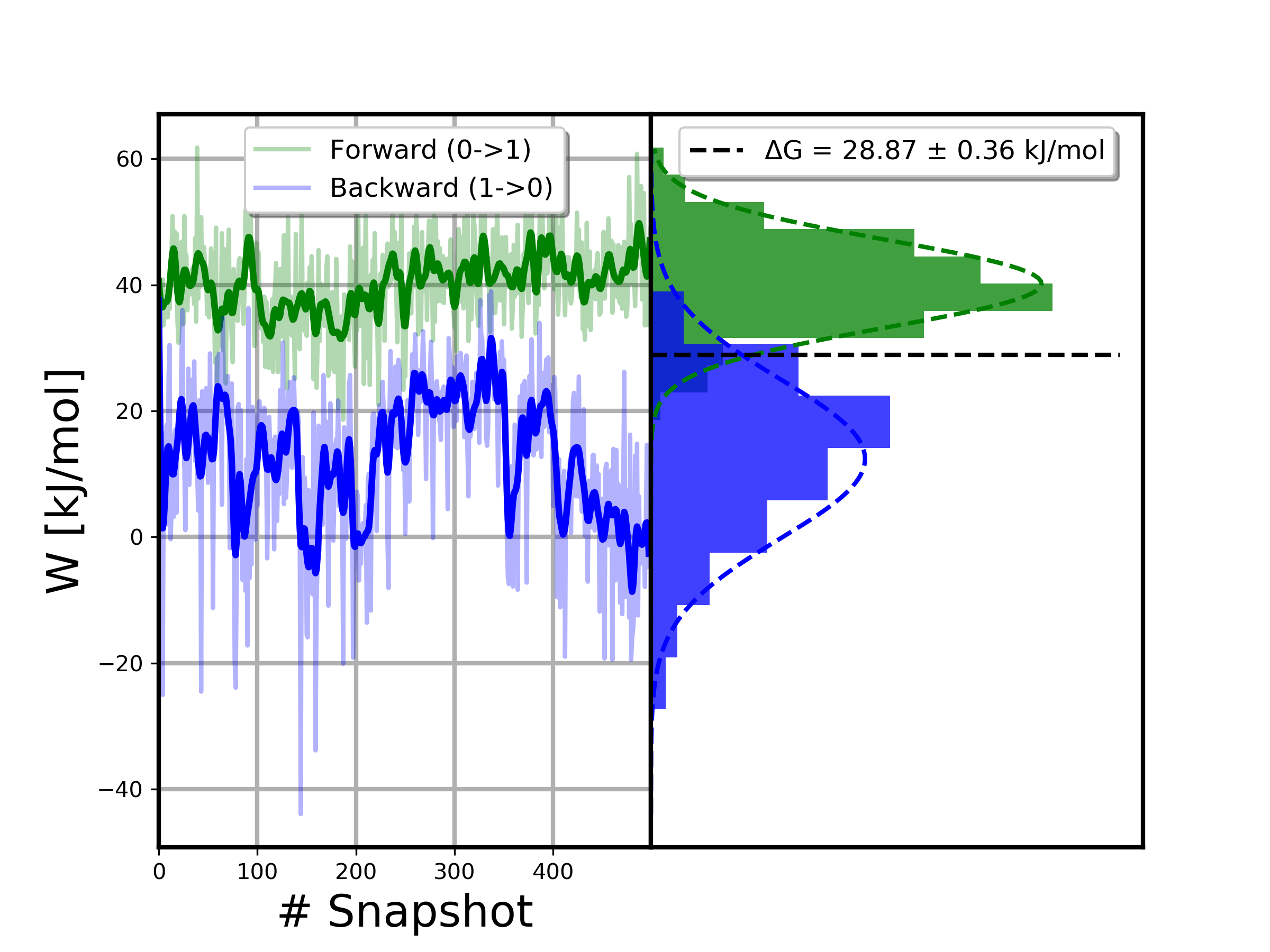

Supplement: Supplementary file 2 [file DataSheet2.ZIP › Raw Date for Redox potential LOXL2-797036/Figure 8 & 10 & Table1/wplot_catalytic-C579-695.png]

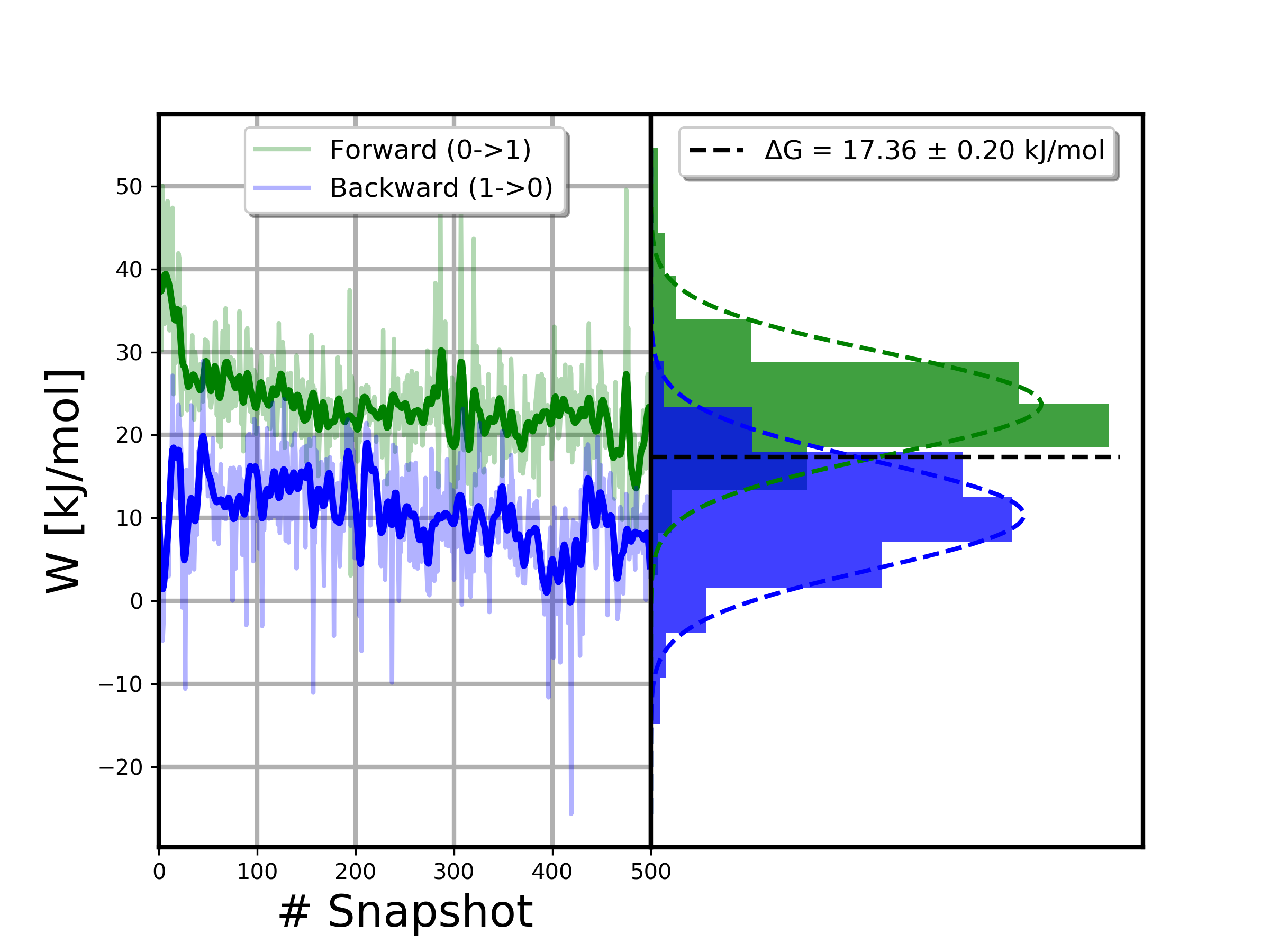

Supplement: Supplementary file 2 [file DataSheet2.ZIP › Raw Date for Redox potential LOXL2-797036/Figure 8 & 10 & Table1/wplot_catalytic-C657-C673.png]

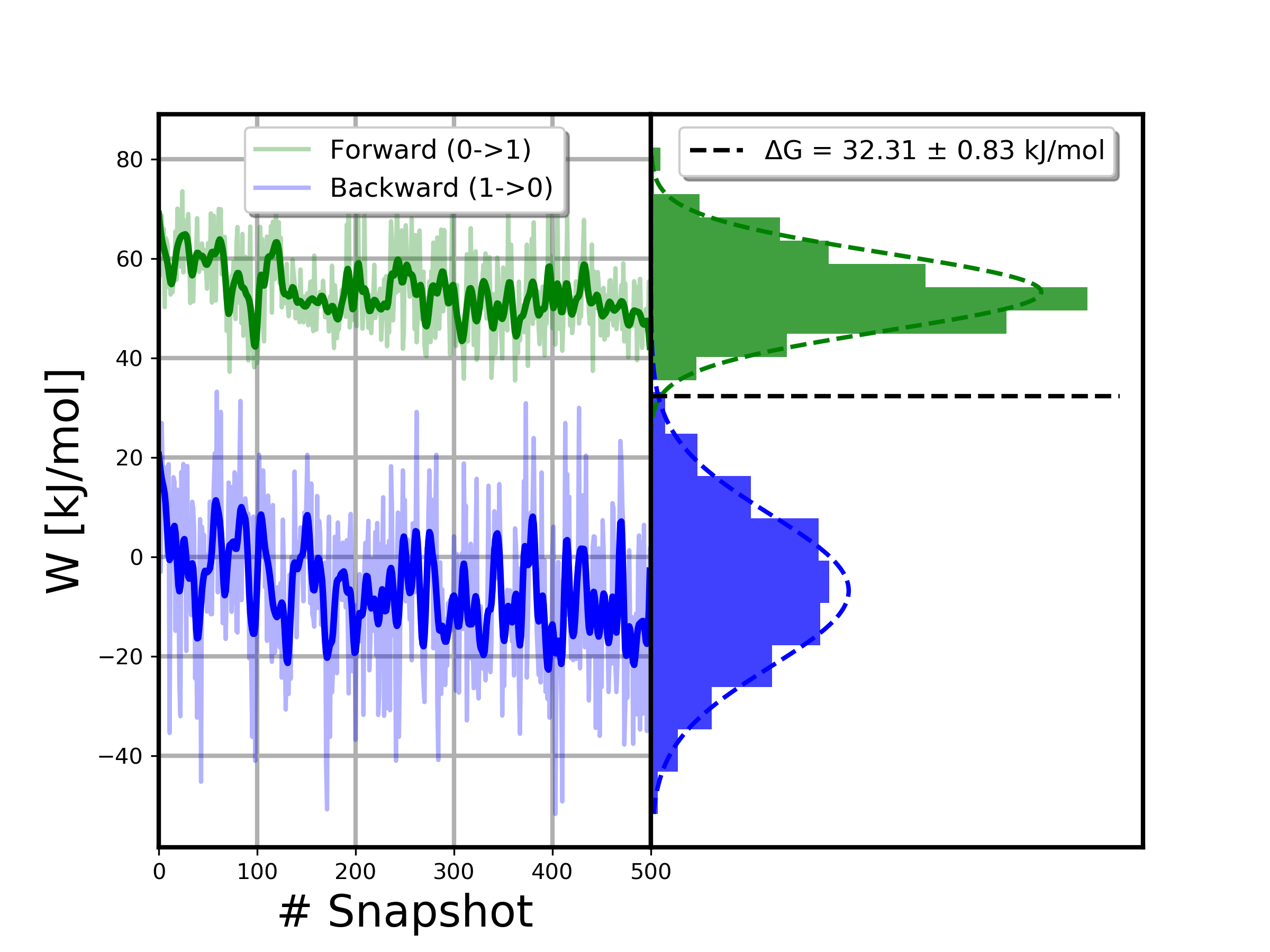

Supplement: Supplementary file 2 [file DataSheet2.ZIP › Raw Date for Redox potential LOXL2-797036/Figure 8 & 10 & Table1/wplot_catalytic-C663-C685.png]

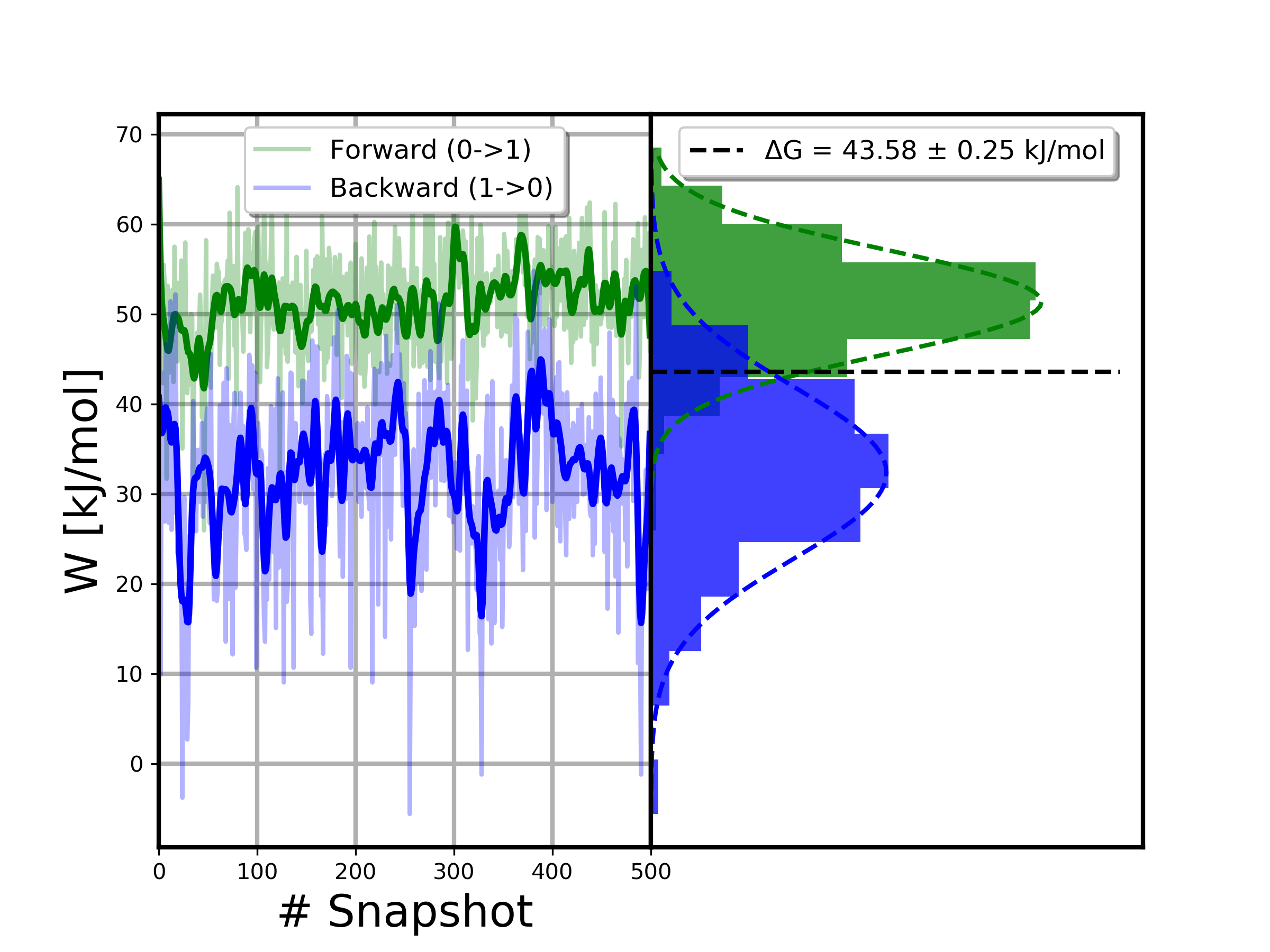

Supplement: Supplementary file 2 [file DataSheet2.ZIP › Raw Date for Redox potential LOXL2-797036/Figure 8 & 10 & Table1/wplot_catalytic-C732-C746.png]
